# The PA-X host shutoff site 100 V exerts a contrary effect on viral fitness of the highly pathogenic H7N9 influenza A virus in mice and chickens

**DOI:** 10.1080/21505594.2024.2445238

**Published:** 2024-12-28

**Authors:** Xia Chen, Ming Kong, Chunxi Ma, Manyu Zhang, Zenglei Hu, Min Gu, Xiaoquan Wang, Ruyi Gao, Shunlin Hu, Yu Chen, Xiaowen Liu, Daxin Peng, Xiufan Liu, Jiao Hu

**Affiliations:** a Key Laboratory of Avian Bioproducts Development, Ministry of Agriculture and Rural Affairs, Yangzhou, China; b Jiangsu Co-Innovation Center for Prevention and Control of Important Animal Infectious Diseases and Zoonosis, Yangzhou University, Yangzhou, Jiangsu, China; c Key Laboratory of Prevention and Control of Biological Hazard Factors (Animal Origin) for Agri-food Safety and Quality, Ministry of Agriculture of China, Yangzhou University, Yangzhou, China; d Joint International Research Laboratory of Agriculture and Agri-Product Safety, Ministry of Education of China, Yangzhou University, Yangzhou, China

**Keywords:** H7N9 avian influenza virus, PA-X, host shutoff, viral fitness, mice, chickens

## Abstract

Several viruses, including influenza A virus (IAV), encode viral factors to hijack cellular RNA biogenesis processes to direct the degradation of host mRNAs, termed “host shutoff.” Host shutoff enables viruses to simultaneously reduce antiviral responses and provides preferential access for viral mRNAs to cellular translation machinery. IAV PA-X is one of these factors that selectively shuts off the global host genes. However, the specific role of PA-X host shutoff activity in viral fitness of IAV remains poorly understood. Herein, we successfully mapped PA-X 100 V as a novel site important for host shutoff of the H7N9 and H5N1 viruses. By analysing the polymorphism of this residue in various subtype viruses, we found that PA-X 100 was highly variable in H7N9 viruses. Structural analysis revealed that 100 V was generally close to the PA-X endonuclease active site, which may account for its host shutoff activity. By generating the corresponding mutant viruses derived from the parental H7N9 virus and the PA-X-deficient H7N9 virus, we determined that PA-X 100 V significantly enhanced viral fitness in mice while diminishing viral virulence in chickens. Mechanistically, PA-X 100 V significantly increased viral polymerase activity and viral replication in mammalian cells. Furthermore, PA-X 100 V highly blunted the global host response in 293T cells, particularly restraining genes involved in energy metabolism and inflammatory response. Collectively, our data provided information about the intricate role of the PA-X host shutoff site in regulating the viral fitness of the H7N9 influenza virus, which furthers our understanding of the complicated pathogenesis of the influenza A virus.

## Introduction

Currently, influenza A virus (IAV) infection remains a global public concern. Like all viruses, IAV is highly dependent on the host-cell protein synthesis machinery to produce its own proteins. To ensure priority access to the host translation machinery, IAV infection results in a rapid decline in global host protein synthesis in infected cells, a process known as “host shutoff” [1]. Established knowledge has demonstrated that host shutoff activity reorganizes the overall host gene expression profile and paves the way for viruses to overhaul the cell’s biology, antagonize the host's innate immune response, and redirect the translation apparatus to assure the priority of viral protein production [1,2]. Current established knowledge has revealed that IAV mediates host shutoff activity mainly through three mechanisms: (1) the nonstructural protein 1 (NS1) protein blocks host cell processing of cellular pre-mRNA and nuclear export, causing global inhibition of host gene expression [3–5]; (2) the viral RNA-dependent RNA polymerase complex (RdRp) mediates the ubiquitination and degradation of host RNA polymerase II (Pol II), which inhibits RNA transcription [6,7]; (3) and the viral accessory PA-X protein selectively degrades host RNA Pol II transcribed mRNAs and ncRNAs both in the nucleus and cytoplasm of infected cells [8–21], which plays a major role in triggering RNA destruction.

The PA-X protein is a small fusion protein encoded by a + 1-reading frame generated by ribosome frameshifting of the PA gene [8]. Basically, all IAVs can express the PA-X protein [22]. The PA-X protein contributes to the global host shutoff activity [8–21] (Table S1) and involvement in the regulation of viral replication, host immune responses and virulence of various IAV subtypes, including the 1918 H1N1 virus [8], H5N1 avian influenza virus (AIV) [10,23,24], H9N2 AIV [25–27, 2009] pandemic H1N1 virus [24,28–31], swine H1N2 virus [32,33], swine H1N1 virus [34], and canine H3N8 and H3N2 viruses [35]. Furthermore, accumulating studies have identified crucial areas or sites that contribute to the host shutoff activity of the PA-X protein in the 2009 pandemic H1N1 virus [9,12,36], seasonal H1N1 virus [15,36,37], human H5N1 virus [38] and 1918 H1N1 virus [39]. However, currently, there are no studies clearly elucidating the functional connection between PA-X host shutoff activity and viral fitness of IAV.

In this study, based on the sequences in the Global Initiative for Shared Avian Influenza Data (GISAID), we systematically analysed the PA-X polymorphism of the human- and avian-origin H5N1, H5N6, H5N8, H9N2 and H7N9 influenza viruses. We found that PA-X polymorphism at site 100 is quite different between H7N9 influenza virus and the other subtypes. Considering the significance of H7N9 influenza virus in public health and the poultry industry, we therefore investigated the biological function of PA-X 100. As a result, we successfully identified that PA-X 100 V contributed to the shutoff activity of the PA-X protein of both H7N9 and H5N1 AIV in 293T cells. We then further extended the function of PA-X 100 V in regulating the viral replication and virulence of H7N9 AIV both in mice and chickens. We noticed that the host shutoff site PA-X 100 V exhibited different effects on regulating viral replication and pathogenicity in mice and chickens. Further RNA-Seq analysis identified the important role of 100 V in downregulating cellular energy metabolism and inflammatory response-related genes in 293T cells, which may account for itsfunction in regulating viral features. Collectively, our study significantly expands our understanding of how PA-X utilizes the host machinery to redirect global host gene expression to modulate viral fitness and pathogenicity in mammals and poultry. In addition, the novel identified host shutoff site 100 V may provide valuable clues for the discovery of novel antiviral drug targets.

## Results

### PA-X 100 V is more prevalent in other subtype viruses rather than in H7N9 virus

**Figure 1.**
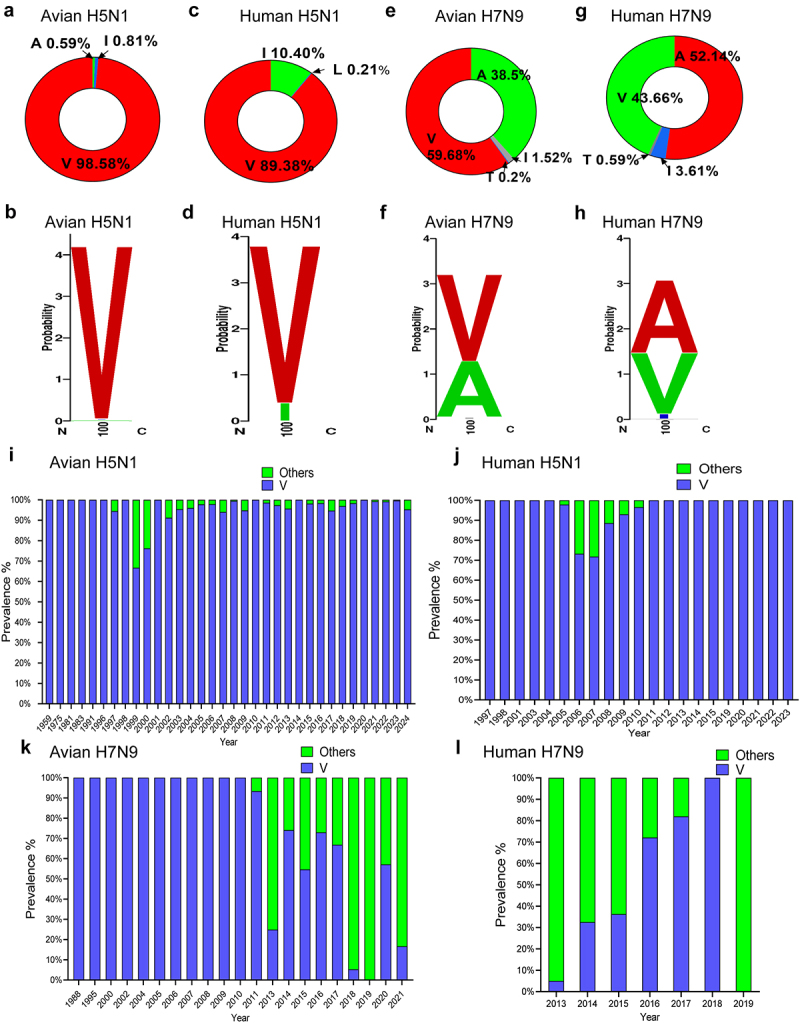
PA-X 100 V is more prevalent in other subtype viruses rather than in H7N9 virus. (a, b) Frequency of PA-X 100 V in avian H5N1 virus by pie (a) or sequence logo (b). (c, d) Frequency of PA-X 100 V in human H5N1 virus by pie (c) or sequence logo (d). (e)-(f) Frequency of PA-X 100 V in avian H7N9 virus by pie (e) or sequence logo (f). (g)-(h) Frequency of PA-X 100 V in human H7N9 virus by pie (g) or sequence logo (h). (i) Frequency of PA-X 100 V in avian H5N1 virus over time. (j) Frequency of PA-X 100 V in human H5N1 virus over time. (k) Frequency of PA-X 100 V in avian H7N9 virus over time. (l) Frequency of PA-X 100 V in human H7N9 virus over time. The sequence logo based on Weblogo was aligned using BioEdit software.

We previously systematically compared all the available PA-X gene sequences of the four prevalent AIV subtypes in China (H5N1, H5N6, H9N2 and H7N9) until 2017 [20]. In this study, we updated the data set by adding the sequences of these subtype viruses until June 2024 and mainly focused on five amino acids in the N-terminal of the PA-X protein. As shown in Table 1, overall, we found that the majority of these sites were highly conserved among different subtypes. However, the PA-X polymorphism of the H7N9 influenza virus at site 100 had a different profile compared to other subtypes. More specifically, PA-X 100 is highly variable both in avian and human H7N9 viruses. We then systematically analysed the frequency of this site in H5N1 and H7N9 subtype viruses. A total of 11,733 avian H5N1 viruses, 471 human H5N1 viruses, 990 avian H7N9 viruses, and 1356 human H7N9 viruses were analysed. Overall, we found that PA-X 100 V was more prevalent in H5N1 subtype virus but not in H7N9 subtype virus (Figures 1a-h). Furthermore, the predominance of PA-X 100 V was quite stable in both avian H5N1 (Figure 1i) and human H5N1 viruses (Figure 1j). Notably, from 2011 to 2023, all the recorded human H5N1 viruses carried the 100 V site. For H7N9 viruses, this residue was stably present in avian H7N9 viruses before 2013, when the Eurasian lineage H7N9 viruses emerged. However, from 2013 to 2021, 100 V was overwhelmed by other residues (Figure 1k). Quite consistent with this, a lower portion of 100 V was also observed in human H7N9 virus during this time period (Figure 1l). Therefore, these bioinformatics data revealed that PA-X 100 V is more prevalent in other subtype viruses rather than in H7N9 virus.

**Table 1.** PA-X polymorphism frequencies in different subtype influenza viruses.

Subtypes/Number of the strains	Frequency of the specific amino acids				
	94I	96N	97T	100 V	115N
H5N1_Avian (total 11,733)	96.39%	99.71%	99.76%	98.58%	98.21%
H5N1_Human (total 471)	62.21%	99.79%	98.94%	89.38%	98.51%
H5N6_Avian (total 1979)	97.62%	99.90%	99.80%	94.64%	97.98%
H5N6_Human (total 45)	100.00%	100.00%	100.00%	77.78%	100.00%
**H7N9_Avian (total 990)**	92.42%	89.47%	99.80%	**59.68%**	89.07%
**H7N9_Human (total 1356)**	97.56%	96.46%	99.41%	**43.66%**	96.16%
H5N8_Avian (total 3368)	99.94%	82.64%	99.94%	92.84%	99.44%
H5N8_Human (total 1)	100.00%	100.00%	100.00%	100.00%	0.00%
H9N2_Avian (total 5225)	99.56%	99.85%	97.61%	86.08%	98.10%
H9N2_Human (total 97)	98.97%	100.00%	97.94%	92.78%	98.97%

### PA-X 100 V contributes to host shutoff activity of H7N9 and H5N1 PA-X proteins in 293T cells

**Figure 2.**
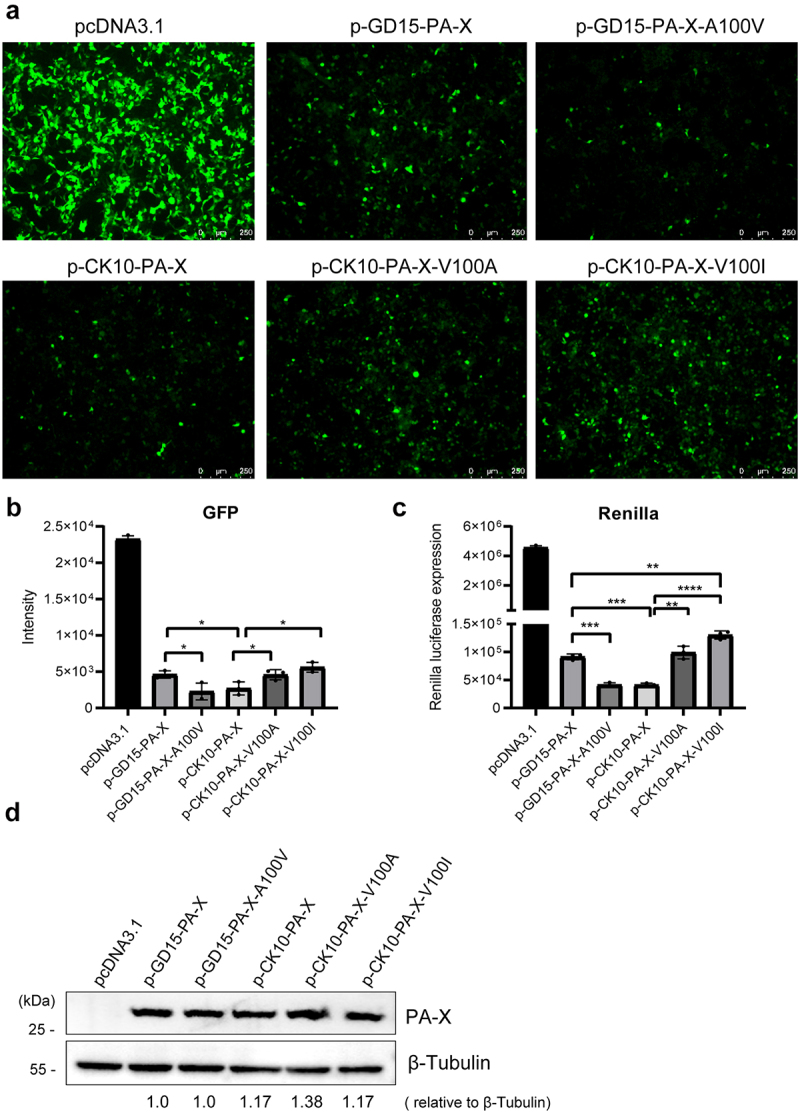
PA-X 100 V contributes to host shutoff activity of H7N9 and H5N1 PA-X proteins in 293T cells. (a) Effect of PA-X protein on the expression of GFP. 293T cells were cotransfected with the pires-hrGFP-1a plasmid encoding GFP and the H7N9 individual PA-X mutant (p-GD15-PA-X, p-GD15-PA-X-A100 V) or the H5N1 individual PA-X mutant (p-CK10-PA-X, p-CK10-PA-X-V100 A, p-CK10-PA-X-V100I) or the pcDNA3.1-flag vehicle. At 48 h post transfection (p.t.), cells were observed under a fluorescence microscope. (b) The intensity of GFP expression in panel a was calculated by image J. (c) Impact of PA-X on the global host gene expression. 293T cells were co-transfected with the individual PA-X plasmids p-GD15-PA-X, p-GD15-PA-X-A100 V, p-CK10-PA-X, p-CK10-PA-X-V100 A, p-CK10-PA-X-V100I or the pcDNA3.1-flag empty vector together with renilla pRL-TK. At 24 h p.t., luciferase production was measured using reagents in the Renilla luciferase reporter assay system. (d) PA-X and cellular β-Tubulin protein expression levels in panel 2c were analysed by western blot using cell extracts and antibodies specific to the flag-tag (to detect PA-X protein) and β-Tubulin. Western blots were quantified by image J. Data were shown as the mean ± standard deviation (SD) of the representative results from three independent experiments.

To evaluate the biological function of PA-X 100 V, we first determined its effect on host shutoff activity of PA-X protein by measuring GFP and pRL-TK expression in 293T cells. As shown in Figure 2, for H7N9 virus (GD15), the inhibitory effect of the parental p-GD15-PA-X on GFP and pRL-TK expression was significantly augmented by the mutant p-GD15-PA-X-A100 V, suggesting a clear contribution of 100 V in the host shutoff activity of H7N9 virus. As for H5N1 virus (CK10), PA-X V100 A or PA-X V100I mutation all significantly weakened the host shutoff ability of PA-X, indicating that 100 V also contributes to the host shutoff efficiency of H5N1 PA-X. Interestingly, compared with the wild-type H7N9 PA-X, the wild-type H5N1 PA-X had a stronger shutoff activity (Figures 2a-c). Meanwhile, when determining the PA-X expression level in Figure 2 panel c, we found a similar expression pattern of PA-X between the parental PA-X protein and the mutant PA-X protein (Figure 2d). Therefore, these results clearly demonstrated that 100 V contributes to the host shutoff efficiency of the H7N9 and H5N1 PA-X proteins in 293T cells.

### PA-X 100 V significantly enhances viral fitness of H7N9 virus in mice

**Figure 3.**
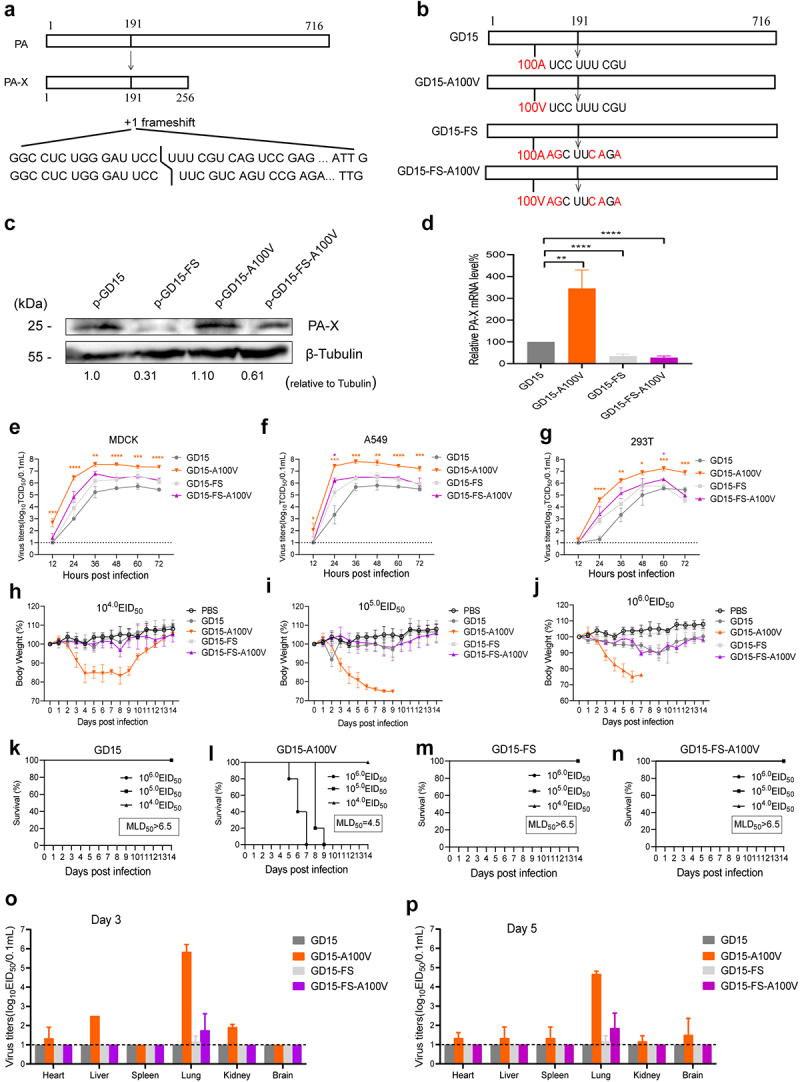
PA-X 100 V significantly enhances viral fitness of H7N9 virus in mice. (a) Schematic representation of PA and PA-X proteins synthesized from the segment 3 transcripts. Continued ORF translation produces full-length viral polymerase subunit PA, while + 1 frameshifiting at 191 codon produces smaller PA-X protein. (b) Three H7N9 recombintant viruses based on the parental GD15 virus were generated, including the PA and PA-X A100 V mutant virus (named GD15-A100 V), the PA-X- deficient virus (named GD15-FS) and the corresponding mutant PA-X- deficient virus carrying only the PA A100 V mutation (named GD15-FS-A100 V). (c) The protein expression levels of PA-X and cellular β-tubulin in panel c. Cells were collected and analysed by western blot using cell extracts and antibodies specific to PA-X mouse polyclonal antibody (prepared in our lab) and β-tubulin. Western blots were quantified by image J. (d) The expression levels of PA-X mRNA by viral infection. MDCK cells were infected with the indicated viruses at a MOI of 2. At 24 h post transfection (p.t.), cells were collected and analysed by qRT-PCR. (e)-(g) Effect of PA-X 100 V on viral replication in MDCK (e), A549 (f) and 293T cells (g). Different cells were inoculated at a multiplicity of infection (MOI) of 0.01 of the indicated viruses. Virus titres were determined as TCID_50_ in MDCK cells at the indicated time points. The data was represented as one of the three independent experiments and shown as the mean ± SD of three independent infections. (h)-(j) Body weight change of the infected mice. Body weight was presented as percentage of the weight on the day of inoculation (day 0). Mice were humanely killed when they lost > 25% of their initial body weight. Mice were infected with a dose of 10^4.0^ EID_50_ (h), 10^5.0^ EID_50_ (i) or 10^6.0^ EID_50_ (j). (k)-(n) Survival rate of the virus-infected mice. Mice were infected with parental GD15 (k), or the mutant GD15-A100 V (l), the PA-X- deficient virus GD15-FS (m) or the mutant GD15-FS-A100 V virus (n). (o)-(p) Effect of 100 V on viral replication in mice. Groups of mice were infected with the indicated recombinant virus. Three mice of each group were euthanized on days 3 (o) and 5 (p) p.t. For determination of viral load in mice infected with 10^6.0^ EID_50_ of the viruses. For statistical analysis, orange “*” means significant difference between GD15-A100 V and the parental GD15 virus, purple “*” represents for significant difference between GD15-FS-A100 V and the parental GD15-FS virus.

PA-X plays an important role in regulating viral fitness and virulence of IAV [20]. To investigate whether the host shutoff site PA-X 100 V affects viral replication, we then subsequently generated PA and PA-X A100 V mutant viruses based on the parental GD15 virus, and the resulting virus was named GD15-A100 V (Figures 3a,b). We also rescued the PA-X- deficient virus based on the wild-type (wt) GD15 virus (named GD15-FS) and the corresponding mutant PA-X- deficient virus carrying only the PA A100 V mutation (named GD15-FS-A100 V). To determine the effect of these modifications on PA-X expression, the indicated PA proteins were transfected to 293T cells. As a result, PA A100 V mutation had no significant effect on PA-X protein level (Figure 3c). Meanwhile, mutation of frame shifting sites resulted in a significant decrease in PA-X mRNA and protein level (Figure 3c). During virus infection, a similar PA-X mRNA pattern was observed as that of PA plasmid (Figure 3d). The growth curves of these viruses were then measured in canine MDCK, human A549, and 293T cells. The results showed that the GD15-A100 V mutant virus exhibited a clear replication advantage over the wt GD15 virus in all these cell types at multiple time points (Figures 3e-g). In contrast, compared with its parental PA-X- deficient virus GD15-FS, the mutant GD15-FS-A100 V only showed a significantly higher replication efficiency in A549 cells at 24 h p.i. and in 293T cells at 60 h p.i. Regarding viral virulence in mice, mice infected with the mutant GD15-A100 V virus displayed more weight loss than the parental GD15 virus-infected mice at infection doses of 10^4.0^, 10^5.0^, and 10^6.0^ 50% embryo infectious dose (EID_50_) (Figures 3h-j). Moreover, some of the GD15-A100 V virus-infected mice lost more than 25% of their initial weight and eventually succumbed to death, while no mortality was observed for the mice infected with the parental or the PA-X- deficient viruses. As a result, the 50% of the mouse lethal dose (MLD_50_) for GD15-A100 V was 4.375 log_10_ EID_50_, while the values for the GD15, GD15-FS, and GD15-FS-A100 V viruses were all higher than 6.5 log_10_ EID_50_ (Figures 3k-n). Therefore, these results suggested that PA-X A100 V mutation significantly enhanced viral replication *in vitro* and obviously increased viral virulence in mice, while the PA A100 V mutation may have no obvious effect on these aspects. Since the PA-X A100 V mutation enhanced viral replication *in vitro*, we next investigated the impact of this site on viral replication of H7N9 virus in mice. The wt GD15 virus failed to recover from any of the organs of the infected mice (Figures 3o,p). In contrast, the corresponding GD15-A100 V mutant virus gained the ability to disseminate from the mouse lung to other organs, including the heart, liver, spleen, kidney, and brain. Meanwhile, the PA-X- deficient viruses GD15-FS and GD15-FS-A100 V could only recover from the mouse lung, and no significant difference was observed in terms of viral titers in mouse lung between these two groups.

**Figure 4.**
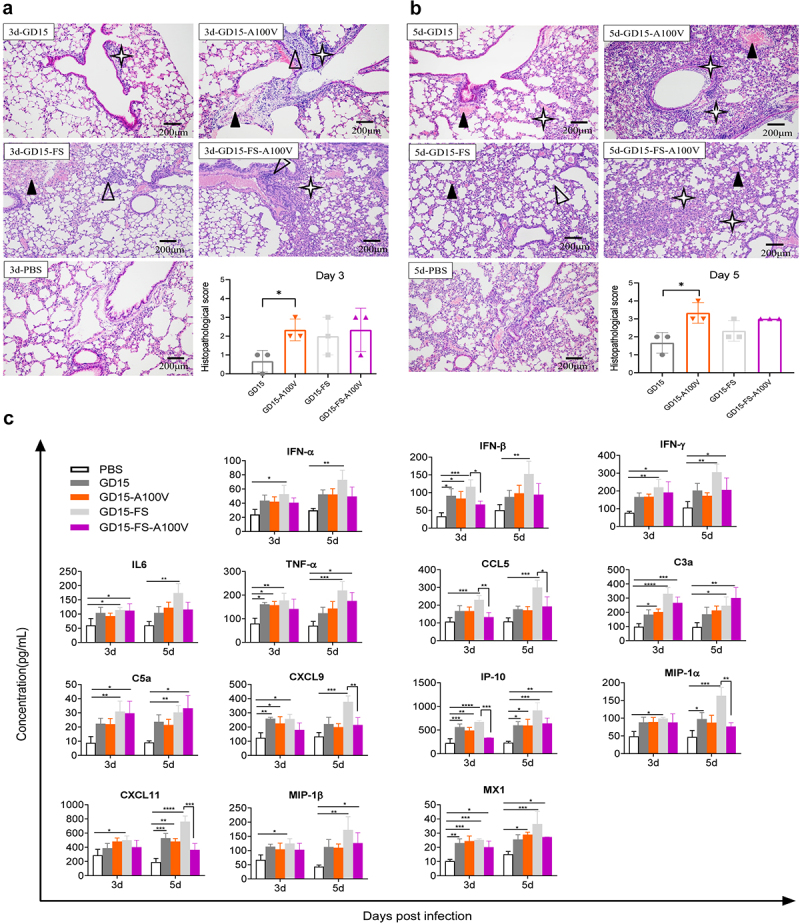
Effect of PA-X 100 V on H7N9 virus-induced histopathological and innate immune response in mice. (a-b) Histopathology and scores of the histopathological changes in the mouse lung-infected with the indicated virus in a dose of 10^6.0^ EID_50_ on day 3 (a) and 5 (b) p.t. 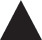, lung congestion and haemorrhage; 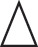, necrotic detached cells were seen in the bronchiolar lumen; 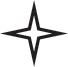, lymphocyte infiltration in pulmonary alveoli bronchiolar lumen and around blood vessel. (c) Innate immune response in mice. Groups of mice were infected with each of the indicated recombinant virus at a dose of 10^6.0^ EID_50_. Three mice of each group were euthanized on day 3 and 5 p.t. For determination of cytokine response in mouse lung. The concentration of cytokines/chemokines and complements derived components in mouse lung was analysed by ELISA. Values were shown as the means ± SD of three samples.

Histopathological analysis of the mouse lung demonstrated that the pathological damage induced by the mutant GD15-A100 V virus was more severe than that induced by the wt GD15 virus at days 3 and 5 p.i. (Figures 4a,b). More specifically, at these time points, the GD15-A100V virus-infected mice showed severe lung congestion and haemorrhage and substantial lymphocyte infiltration in the pulmonary alveoli bronchiolar lumen and around blood vessels. However, for the PA-X- deficient viruses, no significant difference was observed between the GD15-FS and GD15-FS-A100 V virus-infected mice. Therefore, these results revealed that the PA-X A100 V mutation significantly enhanced viral-induced lung injury and markedly strengthened viral replication in mice, while the PA A100 V mutation may have no significant effect on these facets.

To determine whether PA-X 100 V affects virus-induced innate immune responses *in vivo*, the expression profile of a total of 14 cytokine- or complement-derived genes was investigated in these mouse lungs. Overall, compared with mock control, virus infection stimulated a significantly higher expression of these genes (Figure 4c). However, strikingly, although the mutant GD15-A100 V virus replicated significantly higher viral titres in mouse lung than that of the parental GD15 virus, their ability to induce cytokine responses was comparable, suggesting a possible role of PA-X A100 V in decreasing the expression of these genes. Meanwhile, when reducing the expression of PA-X protein, the PA-X-deficiency virus GD15-FS stimulated higher expression level of these cytokines, indicating a possible role of PA-X in shutoff of these genes. Accompanied by this, compared with the wild-type virus GD15-FS, the mutant virus GD15-FS-A100 V significantly hindered the expression levels of IFN-β, CCL5, CXCL9, IP-10, CXCL11, and MIP-1α in mouse lungs, further proving the role of 100 V in host shutoff. Altogether, these results clearly revealed that PA-X A100 V mutation obviously enhanced viral replication and viral fitness in mice.

### PA-X 100 V obviously restrains viral fitness of the H7N9 virus in chickens

**Figure 5.**
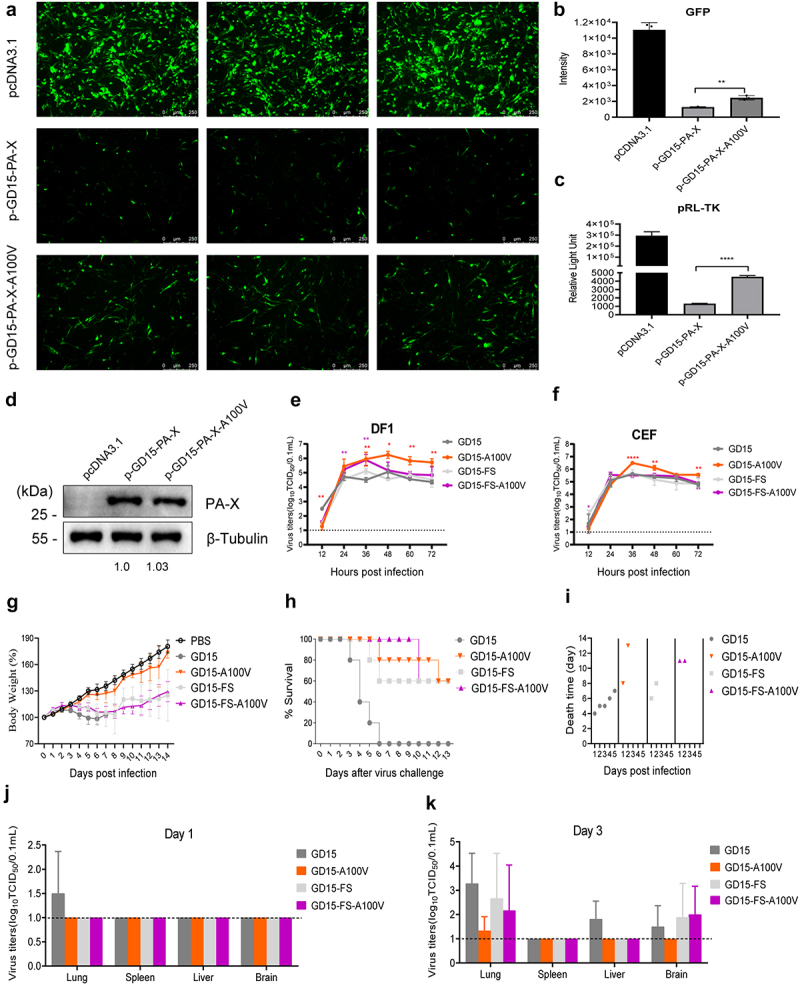
PA-X 100 V obviously restrains the viral fitness of the H7N9 virus in chickens. (a) Effect of PA-X 100 V on GFP expression in DF-1 cells. Cells were cotransfected with pires-hrGFP-1a plasmid which expressing GFP and the individual PA-X mutant or the pcDNA3.1-flag vehicle. At 48 h p.t., cells were observed under a fluorescence microscope. (b) The intensity of GFP in panel a were calculated by image J. (c) Impact of PA-X 100 V on *Renilla* pRL-tk expression in DF-1 cells. Cells were cotransfected with pRL-TK and the indicated PA-X expression plasmids or the pcDNA3.1 vehicle. After 48 h p.t., luciferase production was measured using reagents in the *Renilla* luciferase reporter assay system. (d) PA-X and cellular β-Tubulin protein expression levels in panel 5c were analyzed by western blot using cell extracts and antibodies specific to the flag-tag (to detect PA-X protein) and β-Tubulin. Western blots were quantified by image J. (e)-(f) Effect of PA-X 100 V on viral replication in DF-1 (e) and CEF (f) cells. Different cells were inoculated at a multiplicity of infection (MOI) of 0.01 of the indicated viruses. Virus titers were determined as TCID_50_ in MDCK cells at the indicated time points. The data was represented as one of the three independent experiments and shown as the mean ± SD of three independent infections. (g)-(i) Groups of 5-week-old chickens were infected with the indicated recombinant virus at a dose of 10^6.0^ EID_50_. (g) Body weight of the infected birds. Body weight was presented as percentage of the weight on the day of inoculation (day 0). Chickens were humanely killed when they lost > 25 % of their initial body weight. (h) Survival rate of the infected birds. (i) Death time of the birds. (j)-(k) Viral loads in birds. Three birds of each group were euthanized on day 1 (j) and 3 (k) p.t. For determination of viral load *in vivo.*

Since PA-X 100 V contributed to the host shutoff activity of the PA-X protein in 293T cells, we then asked whether this site affects this aspect in DF-1 cells. Surprisingly, by contrast, we found that PA-X 100 V decreased the host shutoff activity of PA-X in DF-1 cells (Figures 5a-d). We then compared the viral growth curves in DF-1 and CEF cells, and found that PA-X A100 V mutation also enhanced viral replication in these cells (Figures 5e,f). We then further determined whether this site has an impact on viral fitness in chickens. The wt GD15 virus-infected chickens started to show severe disease signs at day 4 p.i., such as weight loss (Figure 5g), cyanosis, oedema of the head and comb, and oedema and red discoloration of the shanks and feet due to subcutaneous ecchymotic haemorrhages, and all birds died in one week (Figures 5h,i). In contrast, only two birds infected with the mutant GD15-A100 V exhibited severe clinical symptoms and eventually died, and the remaining birds developed mild clinical signs, such as ruffled feathers and transient depression, and then recovered and started to gain weight. For the PA-X- deficient viruses, both the parental GD15-FS virus and the mutant GD15-FS-A100 V virus killed only two birds. When comparing viral replication in birds, the parental GD15 virus was successfully recovered from the lung, liver, and brain at day 3 p.i. In contrast, the GD15-A100 V mutant virus could only be detected in the lung at day 3 p.i., correlating with its attenuation in chickens (Figures 5j,k). Meanwhile, both PA-X- deficient viruses can be recovered from the lungs and brains of the infected birds at day 3 p.i. However, there was no significant difference between these two groups.

**Figure 6.**
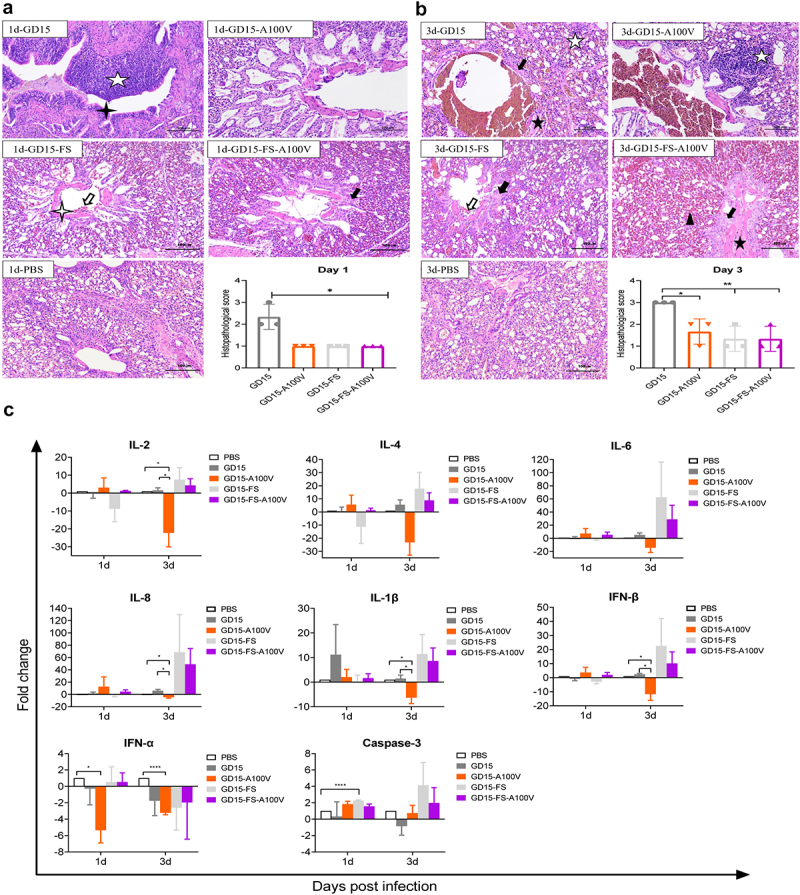
Effect of PA-X 100 V on H7N9 virus-induced histopathological and innate immune response in chickens. (a-b) Histopathology and scores of the histopathological changes in chicken lungs-infected with the indicated virus in a dose of 10^6.0^ EID_50_ on day 1 (a) and 3 (b) p.t. 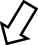, lung haemorrhage; 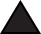, lung congestion; 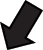, dilation of parabrochus and structure disappearance of the lung chamber; 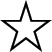, lymphocyte infiltration in blood vessels or parabrochus and/or pulmonary chamber; 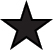, eosinophilic material can be seen in the cavity; 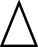, the structure of the exhale capillary was unclear; 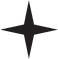, water degeneration, cell swelling and cytoplasm light dye of the epithelial cells in bronchus; 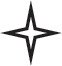, a small amount of epithelial cells was lost in the accessory bronchus. (c) Groups of chickens were infected with each of the indicated recombinant virus at a dose of 10^6.0^ EID_50_. Three chickens of each group were euthanized on day 1 and 3 p.t. for the determination of cytokine response in chicken lungs. The concentration of cytokine/chemokine and complement-derived components in chicken lungs was analysed by qRT-PCR. Values were shown as the means ± SD of three samples.

Histopathological analysis and scoring of lung injury revealed that the mutant GD15-A100 V virus significantly attenuated the severity of lung injury compared with that induced by the parental GD15 virus (Figures 6a,b). By contrast, when the PA-X protein was deleted, the parental GD15-FS virus and the mutant GD15-FS-A100 V virus had no difference on viral-induced lung injury. To determine whether PA-X 100 V affects virus-induced innate immune responses in chickens, the expression profile of a total of 8 cytokines was investigated in these bird’s lungs. Consistent with its lower replication in birds’ lung, the mutant GD15-A100 V virus showed a weaker cytokine response, especially for IL-2, IL-8, IL-1β, IFN-α, and IFN-β (Figure 6c). Meanwhile, overall, at day 3 p.i., compared with the parental GD15 virus, the PA-X-deficiency virus stimulated higher expression level of these cytokines, indicating a potential role of PA-X in shutoff of these genes. However, there was no significant difference between the wild-type GD15-FS virus and the mutant GD15-FS-A100V virus. Collectively, these results clearly revealed that PA-X A100 V substantially dampened viral replication and virulence in chickens, while the PA A100 V mutation may have no obvious effect on these aspects.

### PA-X 100 V markedly enhances viral polymerase activity but has no effect on nuclear accumulation of PA and NP proteins in mammalian cells

**Figure 7.**
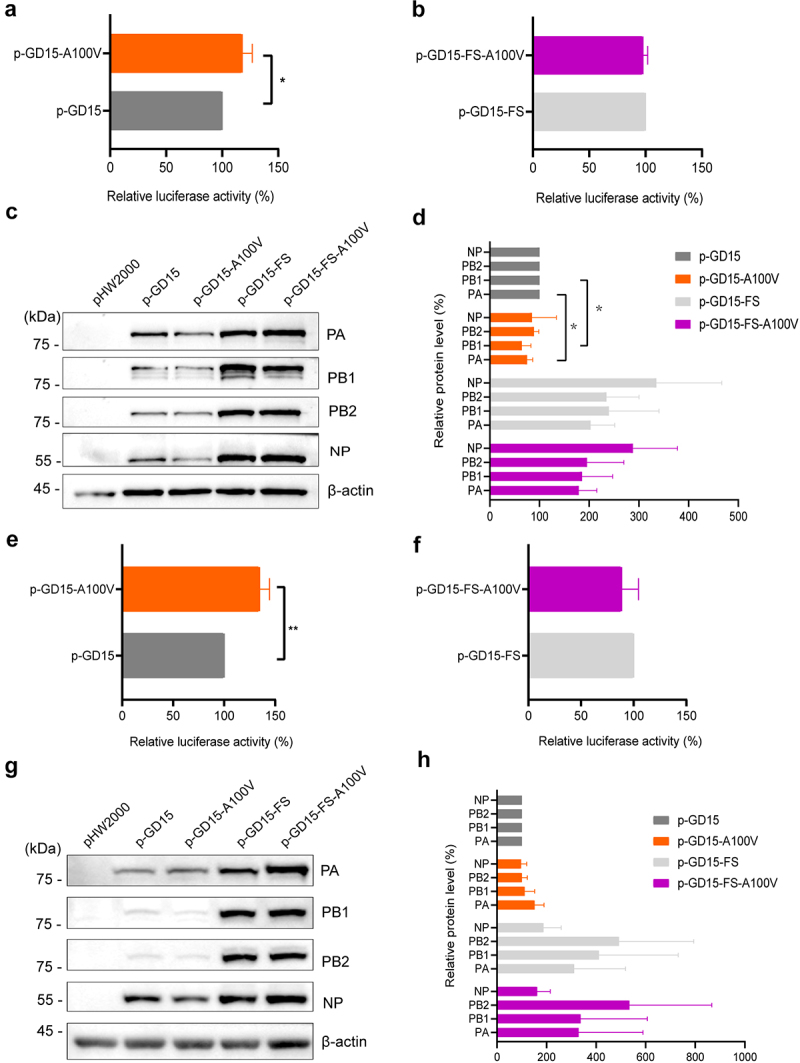
PA-X 100 V enhances viral polymerase activity both in 293T cells and DF-1 cells. 293T cells and DF-1 cells were transfected in triplicate with luciferase reporter plasmid *p*-Luci and internal control plasmid *Renilla* pRL-tk, together with plasmids expressing PB2, PB1, NP, PA or the mutant PA from GD15 virus. At 24 h p.t., cell lysates were used to measure firefly and *Renilla* luciferase activities. Values are shown as the means ± SD of the representative results from three independent experiments and are standardized to those of parental p-GD15 (100%) or p-GD15-FS (100%). (a) Polymerase activity of the parental PA protein p-GD15 and mutant protein p-GD15-A100 V in 293T cells; (b) polymerase activity of the parental p-GD15-FS and mutant p-GD15-FS-A100 V in 293T cells; (c) the expression of PB2, PB1, PA and NP proteins in panel a and panel b were determined by western blotting; (d) the relative expression level of PB2, PB1, PA and NP in panel c were normalized to *β*-actin. (e) Polymerase activity of the parental p-GD15 and mutant p-GD15-A100 V in DF-1 cells; (f) polymerase activity of the parental p-GD15-FS and mutant p-GD15-FS -A100 V in DF-1 cells; (g) the expression of PB2, PB1, PA and NP proteins in panel e and panel f were determined by western blotting; (h) the relative expression level of PB2, PB1, PA and NP in panel g were normalized to *β*-actin.

**Figure 8.**
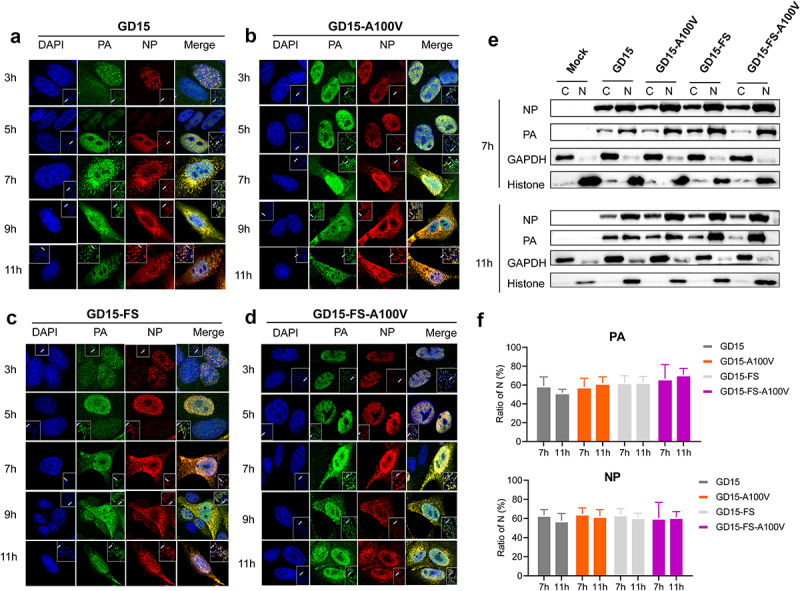
PA-X 100 V has no significant effect on PA and NP nuclear accumulation of H7N9 virus in MDCK cells. (a-d) MDCK cells were infected with GD15 (a), or GD15-A100 V (b) or GD15-FS (c) or GD15-FS-A100 V (d) at a MOI of 2, cell cultures were then fixed and processed for immunofluorescence observation at the indicated time points using anti-pa or NP antibodies. Cell nuclei were stained with DAPI (4,’6-diamidino-2-phenylindole). (e) MDCK cells were infected under the same conditions as for immunofluorescence analysis, followed by fractionation at 7 and 11 h p.t. The cells were then separated into the nuclear fraction (designed as N) and the cytoplasmic fraction (designed as c). Each fraction was analysed by immunoblotting for the distribution of the viral proteins, and the purity of the fractions was controlled by blotting for GAPDH and H3 histone proteins. (f) The relative expression level of PA and NP in the nuclear fraction of the infected cells calculated from panel e. Values were normalized to expression levels of the fraction markers ± SD of the representative data from three independent experiments.

Next, we determined whether PA-X 100 V affects viral polymerase activity. As shown in Figure 7a, the activity of the ribonucleoprotein (RNP) complex carrying the A100 V mutation on the PA protein (p-GD15-A100 V) was significantly higher than that of the parental PA (p-GD15) in 293T cells. However, when introducing A100 V mutation to the PA-X-deficient PA protein, the resulting mutant p-GD15-FS-A100 V had comparable viral polymerase activity than that of the parental p-GD15-FS in 293T cells (Figure 7b). Therefore, these results revealed that only PA-X 100 V significantly affects viral polymerase activity in 293T cells. In addition, when the PA-X protein was retained, a 100 V mutation significantly decreased the PA and PB1 protein expression (Figures 7c,d). Meanwhile, it was obvious that loss of PA-X expression enhanced the amount of RNP complex components, suggesting a possible role of PA-X in shutting off the expression of viral own proteins. However, this may not reflect the real situation during virus infection in host cells. As for the results in DF-1 cells, interestingly, consistent with the results of viral replication *in vitro*, PA-X A100 V mutation also enhanced viral polymerase activity while it had no significant effect on the expression of the RNP complex components (Figures 7e-h). Meanwhile, quite in accord with the results in 293T cells, reduction of PA-X expression also increased the amount of RNP complex components. Since PA-X A100 V mutation affects viral replication both *in vitro* and *in vivo*, we then assessed whether this site affects nuclear accumulation of the polymerase components in MDCK cells. However, both IFA and WB results revealed that PA-X 100 V had no obvious effect on PA and NP nuclear accumulation at 3, 5, 7, 9, and 11 h p.i. (Figure 8).

**Figure 9.**
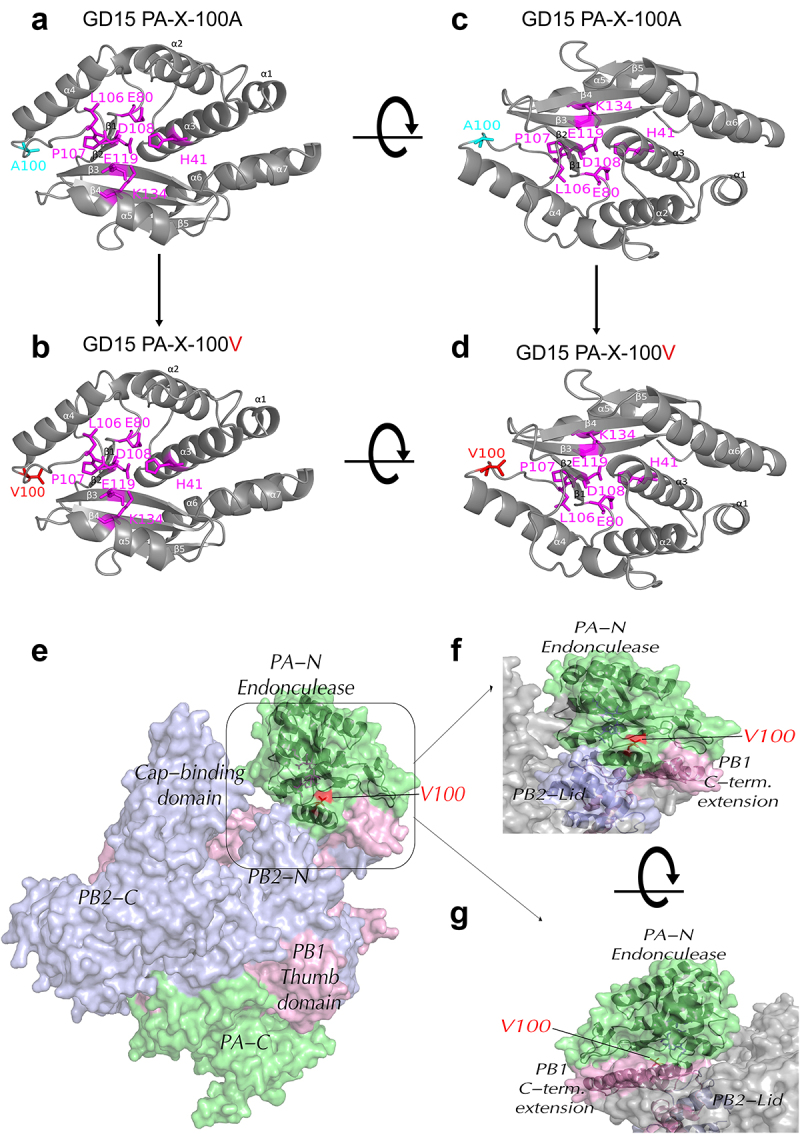
Structural modelling of PA-X 100 V in PA-X protein and assembled polymerase complex. (a-d) the amino acid residues at 100 were mapped onto a ribbon diagram of the structure of the N-terminal region of PA (PDB accession of 2W69.2.A) by SWISS-MODEL (SWISS-MODEL.Expasy.org) using the PyMOL software. Amino acid residues that form the endonuclease active site were shown in magenta. (a and c) the parental PA-X protein GD15 PA-X-100 A; (b and d) the mutant PA-X protein GD15 PA-X-100V. (e-g) Surface diagrams showed the information about the position of PA-X 100 V in the assembled polymerase complex using SWISS-MODEL homology modelling (ProMod3 3.3.0). The PDB: 6evj.2 Cryo-em structure of influenza RNA polymerase was served as the template. Colors used are PA (green), PB2 (blue), PB1 (pink). (e) Schematic of the three subunits showing major domains. (f) Enlarged form of the specific location of PA-X 100 V in assembled polymerase complex. (g) Rotation of the Figure 9f.

To further dissect the function of PA-X 100 V, we then analysed the location of this site in the PA-X protein. Interestingly, PA-X 100 located around the nuclease active site, and compared with site 100 A, site 100 V was generally close to the nuclease active site (Figures 9a-d), suggesting 100 V might structurally support the formation of the nuclease active site. Furthermore, we also analysed the information about the position of PA 100 V in the assembled polymerase complex. We found that this site was close to the PB2-Lid domain and PB1-terminal extension domain (Figures 9e-g), indicating its potential role in regulating viral replication. Altogether, these results clearly showed that the PA-X A100 V mutation enhances viral polymerase activity in 293T cells, which may correlate with its contribution to elevating viral fitness both *in vitro and in vivo*.

### PA-X 100 V exhibits different roles in regulating host response in 293T cells and DF-1 cells

**Figure 10.**
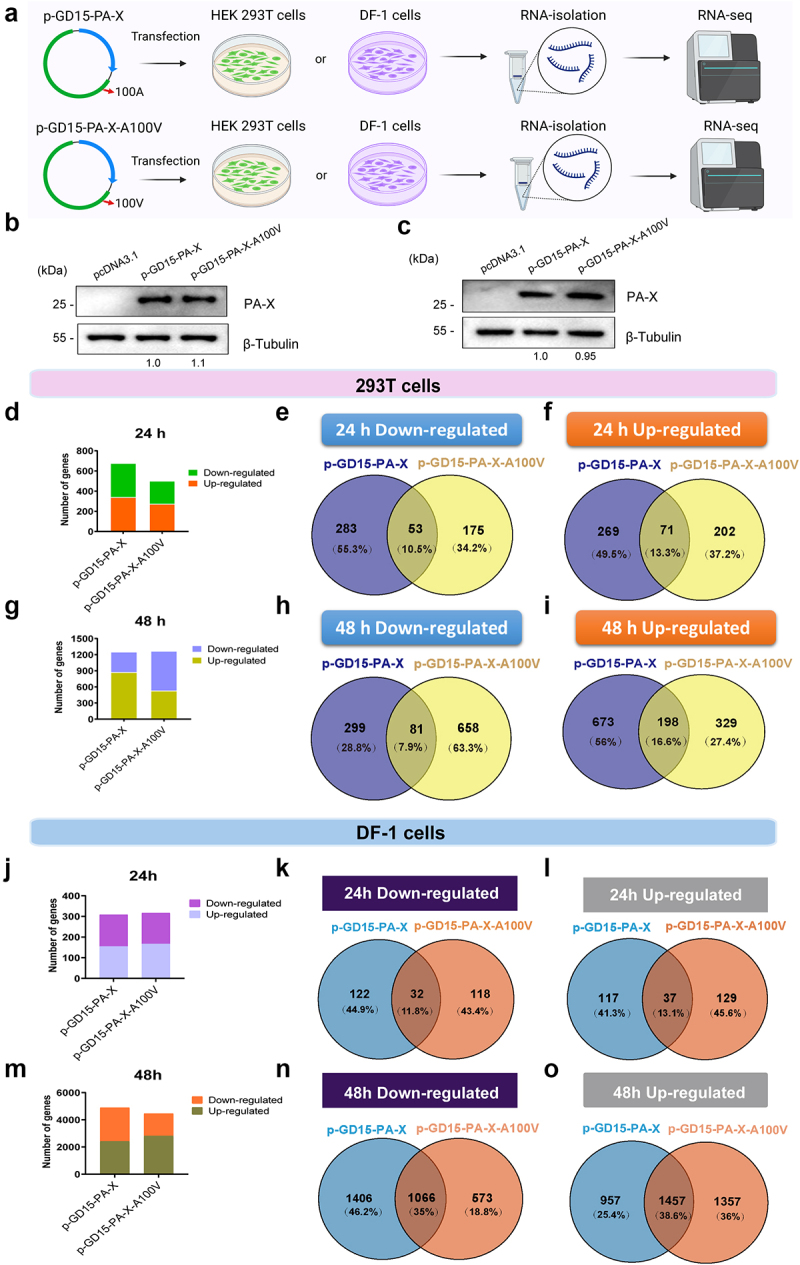
PA-X 100 V exhibits different roles in regulating the global host response in 293T cells and DF-1 cells. (a) The 293T cells and DF-1 cells were either transfected or un-transfected with 2 µg of each plasmid for 24 or 48 h in triplicates. Total RNA was collected for RNA-Seq analysis. (b)-(c) PA-X and cellular β-Tubulin protein expression levels in 293T cells (b) and DF-1 cells (c) in panel a were analyzed by western blot using cell extracts and antibodies specific to the flag-tag (to detect PA-X protein) and β-Tubulin. Western blots were quantified by image J. (d) Numbers of the SDE genes at 24 h p.t. In 293T cells (*p* < 0.05, Fold change > 2). (e)-(f) Venn diagram showing the distribution of the down regulated SDE genes (E) or up regulated SDE genes (f) at 24 h p.t. In 293T cells. (g) Numbers of the SDE genes at 48 h p.t. In 293T cells (*p* < 0.05, Fold change > 2). (h)-(i) Venn diagram showing the distribution of the down regulated SDE genes (H) or up regulated SDE genes (i) at 48 h p.t. In 293T cells. (j) Numbers of the SDE genes at 24 h p.t. In DF-1 cells (*p* < 0.05, Fold change > 1.41). (k)-(l) Venn diagram showing the distribution of the down regulated SDE genes (k) or up regulated SDE genes (l) at 24 h p.t. In DF-1 cells. (m) Numbers of the SDE genes at 48 h p.t. In DF-1 cells (*p* < 0.05, Fold change > 1.41). (n)-(o) Venn diagram showing the distribution of the down regulated SDE genes (n) or up regulated SDE genes (o) at 48 h p.t. In DF-1 cells.

To further investigate the mechanism underlying the functional PA-X 100 V, we then compared the global transcriptomic profile in 293T cells and DF-1 cells induced by the parental PA-X (p-GD15-PA-X) protein or the mutant PA-X (p-GD15-PA-X-A100 V) protein using high-throughput RNA sequencing (RNA-seq) (Figure 10a). A similar PA-X expression level was observed between these two plasmids in both 293T cells (Figure 10b) and DF-1 cells (Figure 10c). However, the parental PA-X protein and the mutant PA-X protein p-GD15-PA-X-A100 V show clear difference in targeting the overall cellular response in 293T and DF-1 cells at different time points.

In 293T cells, screening of the significant differentially expressed (SDE, fold change > 2 and *p* value < 0.01) genes revealed a remarkable difference in the overall transcriptional host response between transfected and non-transfected cells at 24 and 48 h post transfection (p.t.) (Figures 10d-i). More specifically, as shown in Figure 10d, at 24 h p.t., a total of 676 SDE genes were stimulated by the parental PA-X protein, whereas the corresponding number for the mutant protein p-GD15-PA-X-A100 V was 501. In addition, a Venn diagram illustrating the distribution of the SDE genes showed that only a few genes were shared by the two groups (Figures 10e,f). At 48 h p.t., although the parental and mutant proteins stimulated a comparable number of SDE genes (Figure 10g), cells transfected with p-GD15-PA-X-A100 V exhibited a dramatic increase in the number of the down regulated SDE genes (Figures 10h,i). By contrast, the host shutoff activity was relatively stable in p-GD15-PA-X-transfected cells at 24 h p.t. and 48 h p.t. Therefore, these results highlighted the direct role of PA-X 100 V in suppressing global host gene expression, which was consistent with the results of the luciferase reporter gene assay in 293T cells (Figure 2). In addition, a few genes were regulated by the parental p-GD15-PA-X and the mutant p-GD15-PA-X-A100 V (Figures 10h,i) at 48 h p.i., suggesting their great discrepancy in regulating the overall host response.

By contrast, in DF-1 cells, at 24 h p.t., comparable genes were induced by the parental protein p-GD15-PA-X and the mutant protein p-GD15-PA-X-A100 V (the number of SDE genes for p-GD15-PA-X was 308 and for p-GD15-PA-X-A100 V was 316) (Figure 10j). However, at 48 h p.t., relative to p-GD15-PA-X, p-GD15-PA-X-A100 V showed an obvious attenuation in suppressing global host genes (number of down regulated SDE genes for p-GD15-PA-X was 2472, for p-GD15-PA-X-A100 V was 1639) (Figure 10m). The two proteins shared comparable SDE genes at 24 h p.t. in 293T and DF-1 cells, while at 48 h p.t., they shared more SDE genes in DF-1 cells than that in 293T cells (Figures 10k,l,n,o). Altogether, these results revealed that PA-X 100 V exhibits a different role in regulating host response in 293T cells and DF-1 cells, suggesting a possible link for their discrepancy in modulating viral fitness in mice and chickens.

### PA-X 100 V exhibits a remarkable role in reorganizing the global host response in 293T cells

**Figure 11.**
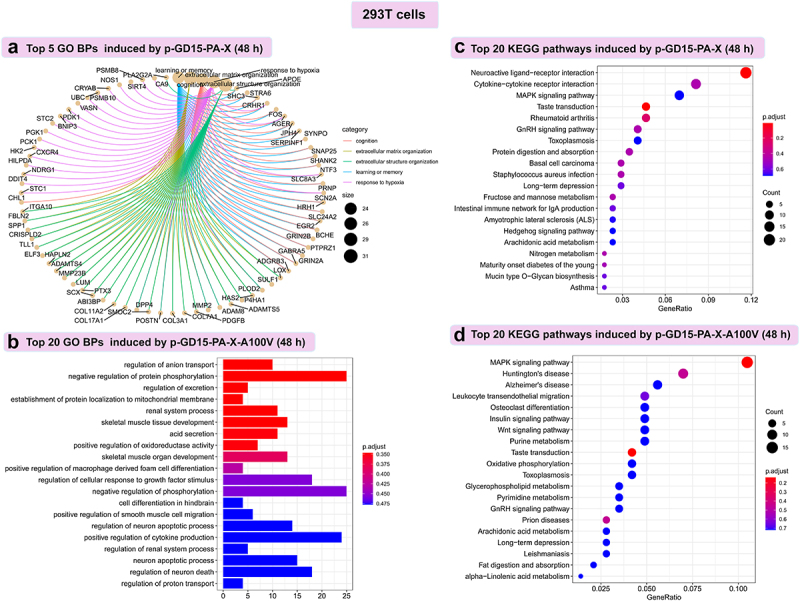
PA-X 100 V exhibits a remarkable role in regulate genes involved in negative regulation of protein phosphorylation and MAPK signalling pathway in 293T cells. (a) Top 5 GO biological process (BPs) induced by the parental PA-X protein p-GD15-PA-X at 48 h p.t. (b) Top 20 GO BPs induced by the mutant PA-X protein p-GD15-PA-X-A100 V at 48 h p.t. (c) Top 20 KEGG pathways induced by the parental PA-X protein p-GD15-PA-X at 48 h p.t. (d) Top 20 KEGG pathways induced by the mutant PA-X protein p-GD15-PA-X-A100 V at 48 h p.t.

Since PA-X 100 V exerted a different role on virus phenotype in mice and chickens, we then compared the primary biological function affected by this site in 293T cells and DF-1 cells. In 293T cells, overall, we found a great discrepancy between the wild-type PA-X protein and the mutant PA-X protein both at 24 h p.t. and at 48 h p.t. More specifically, at 48 h p.t., based on the top Gene Ontology (GO) term analysis, the SDE genes of the parental p-GD15-PA-X were significantly enriched for genes involved in extracellular matrix organization, response to hypoxia (Figure 11a), etc. In contrast, the SDE genes regulated by p-GD15-PA-X-A100 V were primarily associated with the negative regulation of protein phosphorylation and positive regulation of cytokine production (Figure 11b). Further top KEGG pathway analysis revealed that the parental p-GD15-PA-X preferentially regulates genes involved in neuroactive ligand‒receptor interactions (Figure 11C), while the mutant p-GD15-PA-X-A100 V tends to stimulate genes associated with the MAPK signalling pathway (Figure 11d). Distinctive discrepancy in terms of the top GO and KEGG profiles among the PA-X constructs were also observed at 24 h p.t. The SDE genes regulated by p-GD15-PA-X were markedly involved in response to virus, response to type I interferon (Figure S1A), and MAPK signalling pathway (Figure S1B). In contrast, the SDE genes regulated by p-GD15-PA-X-A100V were mainly associated with the negative regulation of cellular carbohydrate metabolic processes (Figure S2A) and cytokine‒cytokine receptor interactions (Figure S2B).

In DF-1 cells, by contrast, great discrepancy was mainly observed at 24 h p.t. At this time point, p-GD15-PA-X tended to regulate system development, transcription by RNA polymerase II (Figure S3A), basal transcription factors, alanine, aspartate and glutamate metabolism (Figure S3B), while p-GD15-PA-X-A100 V was mainly focused on cellular macromolecule and protein localization (Figure S3C), glycosylphosphatidylinositol (GPI)-anchor biosynthesis and protein export (Figure S3D). In contrast, at 48 h p.t., most of the enriched biological function induced by these two proteins overlapped (as indicated by red arrow) (Figure S4). Altogether, these data clearly demonstrated that the sites 100 A and 100 V differ greatly in reorganizing the global host transcriptomic response, especially in 293 T cells.

**Figure 12.**
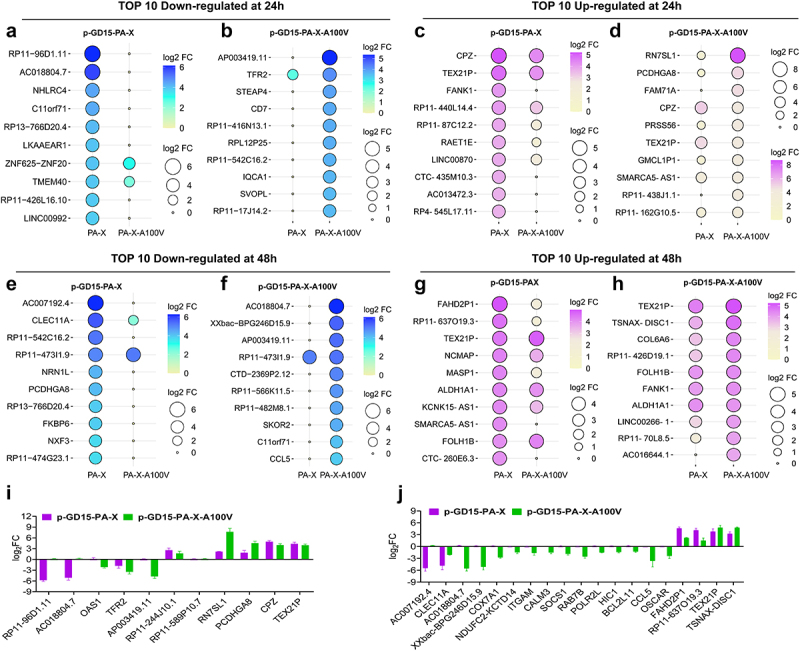
PA-X 100 V shows a substantial role in degrading host genes in 293T cells. (a-b) top 10 down regulated SDE genes regulated by the parental PA-X protein p-GD15-PA-X (a) or the mutant PA-X protein p-GD15-PA-X-A100 V (b) at 24 h p.t. (c)-(d) top 10 up regulated SDE genes regulated by the parental PA-X protein p-GD15-PA-X (c), or the mutant PA-X protein p-GD15-PA-X-A100 V (d) at 24 h p.t. (e-f) top 10 down regulated SDE genes regulated by the parental PA-X protein p-GD15-PA-X (e) or the mutant PA-X protein p-GD15-PA-X-A100 V (f) at 48 h p.t. (g)-(h) top 10 up regulated SDE genes regulated by the parental PA-X protein p-GD15-PA-X (g), or the mutant PA-X protein p-GD15-PA-X-A100 V (h) at 48 h p.t. (i)-(j) qRT-PCR analysis of the selected top-regulated SDE genes regulated by the parental PA-X protein or the mutant PA-X protein at 24 h p.T (I) and 48 h p.t. (j).

Considering the great discrepancy of the sites 100 A and 100 V in reorganizing the global host response in 293T cells, we then compared the expression profiles of the top 10 up or top 10 down regulated genes stimulated by the parental or the mutant PA-X protein in this cell. Strikingly, in terms of the top 10 down regulated genes, the two PA-X proteins showed distinctive differences in suppressing of these genes, suggesting their clear preference for degrading host genes (Figures 12a,b,e,f). In contrast, when analysing the top 10 up regulated genes, these two proteins showed similar trends in regulating these genes, especially at 48 h p.t. (Figures 12c,d,g,h). To further validate these findings, a total of 30 representative RNAs that were strongly down-regulated or up-regulated were selected for the determination of their expression pattern by qRT-PCR. The genes included the top 2 up- and downregulated genes of each group and those associated with important biofunctions, including carboxypeptidase Z (CPZ), 2”-5”-oligoadenylate synthetase 1 (OAS1), chemokine (C–C motif) ligand 5 (CCL5), suppressor of cytokine signalling 1 (SOCS1), BCL2-like 11 (BCL2L11), and integrin subunit alpha M (ITGAM). As a result, the qRT‒qPCR results were quite in agreement with the RNA-seq data in terms of the selective effects on the tested transcripts both at 24 (Figure 12i) and 48 h p.i. (Figure 12j), suggesting a significant role of the host shutoff site 100 V in regulating the global host immune response.

Collectively, these results clearly indicated that PA-X 100 V exhibits a remarkable role in reorganizing the global host response in 293T cells. Notably, PA-X 100 V had an obvious role in degrading host mRNAs in 293T cells. We hypothesized that the different role of 100 V in reshaping the global host immune response in 293T cells and DF-1 cells may partially account for their discrepancy in regulating viral replication and virulence in mice.

### PA-X 100 V strongly and preferentially degrades genes involved in cellular energy metabolism and the inflammatory response in mammalian cells

**Figure 13.**
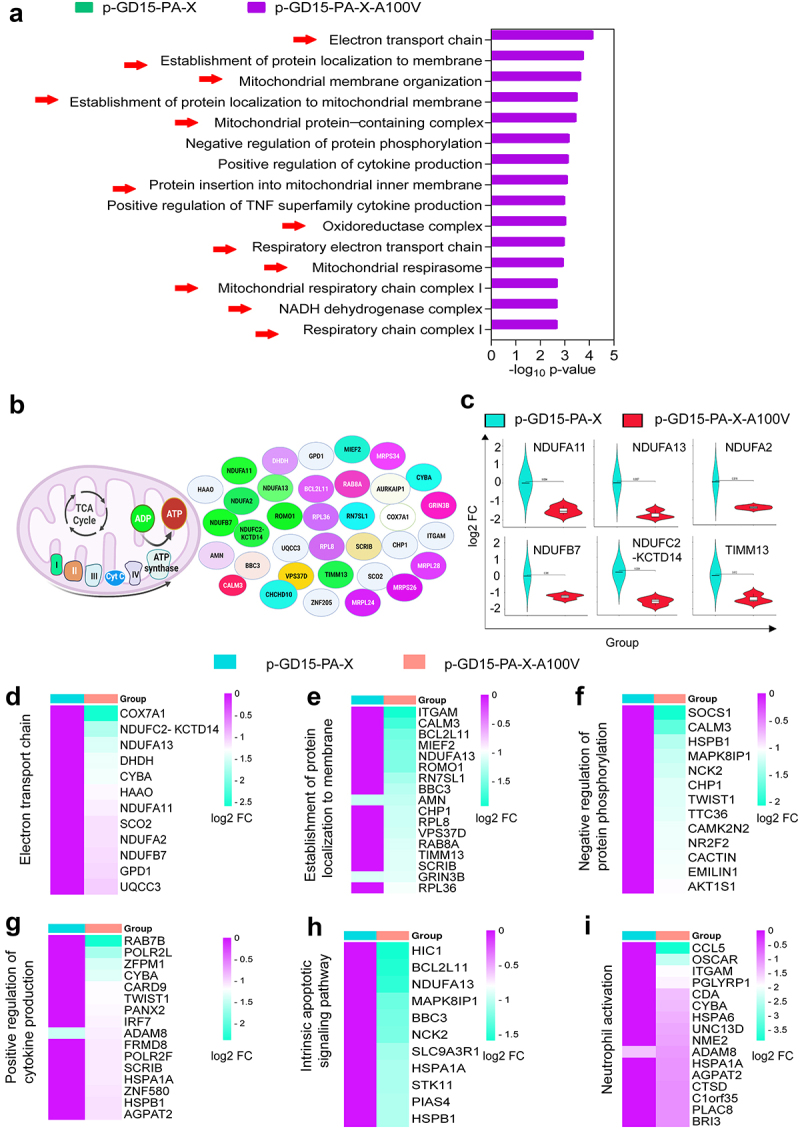
PA-X 100 V strongly and preferentially degrades genes involved in cellular energy metabolism and inflammatory response in 293T cells. (a) The top 15 GO BPs-mediated by the down regulated SDE genes induced by the mutant p-GD15-PA-X-A100 V at 48 h p.t. (b) The energy metabolism related-sde genes that down regulated by the mutant p-GD15-PA-X-A100 V. (c) The expression pattern of the mitochondrial respiratory chain complex I-related genes was verified by qRT‒PCR. (d-i) Heat maps were shown as the gene expression profile of the top GO BPs induced by the mutant p-GD15-PA-X-A100 V. (d) Electron transport chain. (e) Establishment of protein localization to membrane. (f) Negative regulation of protein phosphorylation. (g) Positive regulation of cytokine production. (h) Intrinsic apoptotic signalling pathway. (i) Neutrophil activation.

In 293T cells, at 48 h p.t., we noticed that p-GD15-PA-X-A100V significantly down regulated 739 genes, whereas the corresponding number for the parental p-GD15-PA-X protein was 380 (Figure 10g). To identify the biological function of the down regulated mRNAs targeted by these two proteins, we then analysed the GO terms mediated by these genes. The top 15 GO term analyses’ results demonstrated that the p-GD15-PA-X-A100 V mutant preferentially degraded genes involved in energy metabolism, including genes related to the respiratory electron transport chain, oxidoreductase complex, mitochondrial respiratory chain complex I and mitochondrial membrane function, suggesting its prominent role in restraining cellular energy metabolism (Figures 13a,b; Excel S1). In contrast, the parental p-GD15-PA-X showed a clear preference for destroying genes involved in the adenylate cyclase-modulating G protein-coupled receptor signalling pathway and metabolic process (Figure S5A). The preferential targeting of mRNAs involved in energy metabolism revealed by the GO term analysis may suggest an additional strategy of viruses possessing PA-X 100 V to induce stronger inhibition of host protein synthesis in 293T cells. Since mitochondrial respiratory chain complex I is the largest component of the oxidative phosphorylation system, we then determined the expression pattern of the component of this complex by qRT-PCR in 293T cells. Our findings indicate a significant role of shutoff-active protein PA-X A100 V in suppressing the expression of mitochondrial respiratory chain complex I-related genes in 293T cells, including NDUFA11, NDUFA13, NDUFA2, NDUFB7, NDUFC2-KCTD14, and TIMM13 (Figure 13c).

The significant regulated GO BPs were then further ranked based on the number of involved genes, and the top 6 of these BPs were selected for further analysis. Again, we found a clear difference among these groups. The p-GD15-PA-X-A100 V tended to degrade genes involved in the electron transport chain (Figure 13d), establishment of protein localization to the membrane (Figure 13e), negative regulation of protein phosphorylation (Figure 13f), positive regulation of cytokine production (Figure 13g), intrinsic apoptotic signalling pathway (Figure 13h) and neutrophil activation (Figure 13i). These results again suggest a prominent role of PA-X A100 V in restraining key host genes involved in energy metabolism and the inflammatory response. In contrast, p-GD15-PA-X tended to inhibit genes associated with the adenylate cyclase-modulating G protein-coupled receptor signalling pathway (Figure S5B), organic acid transport (Figure S5C) and extracellular matrix organization (Figures S5D-G), etc. Hence, these data demonstrated that the specific functional classes of host RNAs are differentially sensitive to the parental p-GD15-PA-X and the mutant p-GD15-PA-X-A100 V, highlighting the special role of PA-X 100 V in degrading energy metabolism and inflammatory response-related genes.

**Figure 14.**
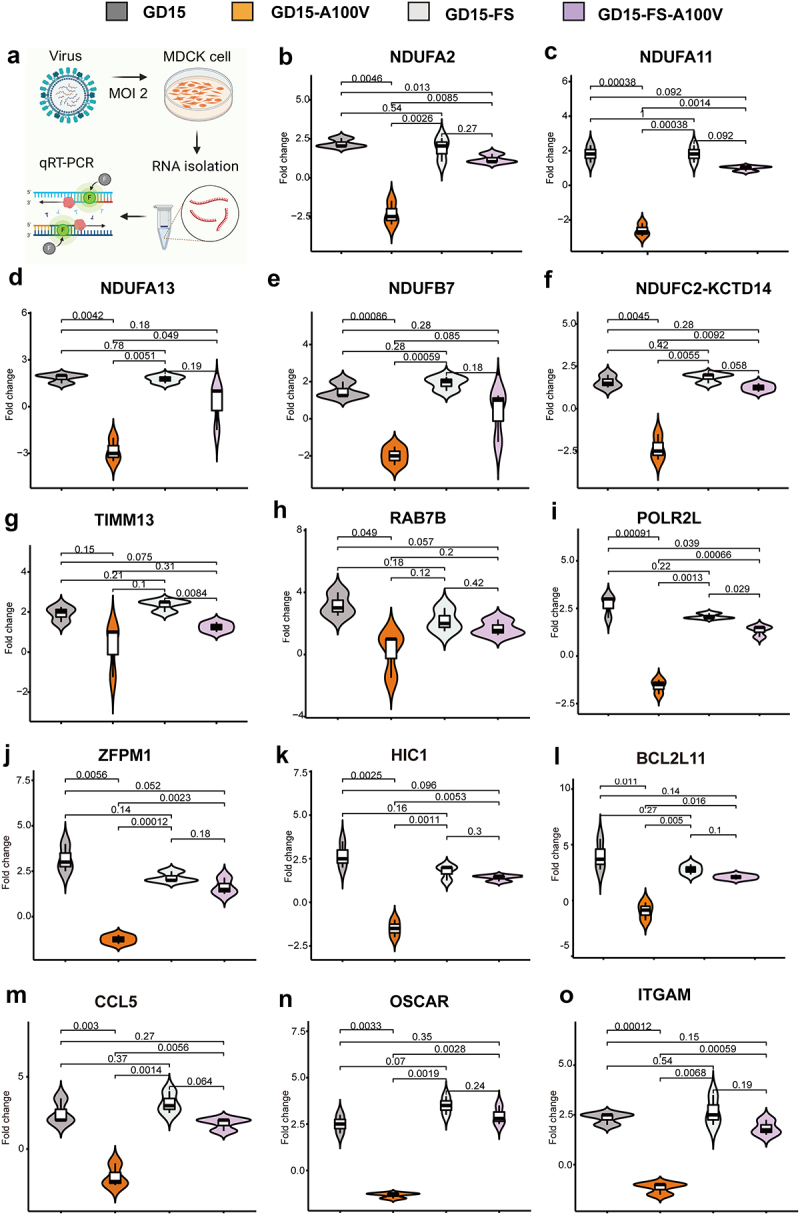
PA-X 100 V shows obvious advantages in suppressing genes involved in cellular energy metabolism signalling pathways and inflammatory response at 48 h p.I. In MDCK cells. MDCK cells were infected with the indicated viruses with a MOI of 2, at 48 h p.I., cells were collected for qRT‒PCR analysis of the cytokines. Among these genes, 6 genes were related to the respiratory electron transport chain (NDUFA11, NDUFA13, NDUFA2, NDUFB7, NDUFC2-KCTD14, TIMM13) and 12 genes were related with inflammatory response (RAB7B, POLR2L, ZFPM1, HIC1, BCL2L11, CCL5, OSCAR, ITGAM). (a) Schematic representation of the qRT-PCR experiments for the virus infection samples. (b-o) Expression pattern of each gene.

To further verify the preferential role of PA-X 100 V in degrading energy metabolism and inflammatory response-related genes, we then compared the expression profile of 14 genes by infection of MDCK cells with different recombinant viruses. Among these genes, 6 genes were related to the respiratory electron transport chain (NDUFA11, NDUFA13, NDUFA2, NDUFB7, NDUFC2-KCTD14, and TIMM13) and 12 genes were related with inflammatory response (RAB7B, POLR2L, ZFPM1, HIC1, BCL2L11, CCL5, OSCAR, and ITGAM). As shown in Figure S6, at 24 h p.i., there were no clear differences among these groups. However, at 48 h p.i., the mutant GD15-A100 V virus showed an obvious advantage in degrading these genes (Figure 14). Therefore, these results directly demonstrated that A100 V mutation markedly increased the ability of PA-X to suppress genes involved in cellular energy metabolism signalling pathways and inflammatory response in mammalian cells.

By contrast, in DF-1 cells, quite similar to the results of the overall host response (Figures S3, S4), major difference between the two proteins was mainly observed at 24 h p.t. More specifically, at 24 h p.t., the downregulated SDE genes of p-GD15-PA-X were mainly enriched for system development (Figure S7A) and MAPK signal pathway (Figure S7B), while p-GD15-PA-X-A100 V was macromolecule localization (Figure S7A) and ECM–receptor interaction (Figure S7C). However, at 48 h p.t., the majority of the enriched biological function-mediated by the down regulated SDE genes that stimulated these two proteins were quite overlapped (as indicated by red arrow) (Figure S8).

Taken together, these results clearly demonstrated that the site 100 V played an important role in directing the PA-X-mediated degradation of host mRNAs, and its particular preferential targeting of mRNAs involved in energy metabolism and the inflammatory response in 293T cells may further explain its functions in regulating viral fitness in mice.

## Discussion

Viral infection induces a wide range of host defence responses, including antiviral and innate immune responses. However, to antagonize the host defence system, viruses can express proteins to evade the innate immune response or to target general host protein synthesis. One of the strategies used by the viruses to suppress host protein synthesis is “host shutoff.” Meanwhile, host shutoff also contributes to counteract host antiviral activity and to redirect the translation apparatus for production of viral proteins. Currently, the NS1 and PA-X of influenza A virus can induce general host shutoff. Since the PA-X protein was first discovered in 2012 [8], accumulated studies have explored its host shutoff function in various subtypes of influenza virus [9,12,15,36–41]. Initially, the endonuclease active sites, residing in the N-terminal domain of PA-X, are identified as responsible for the cellular mRNA degradation [9]. Later, the unique C-terminal region of the PA-X protein was also found to be crucial for host shutoff activity, particularly the six basic amino acids within the N-terminal 15 residues [12,15]. Moreover, other sites important for host shutoff were also discovered [36,37,39–42], including 28P and 65S in 2019 h1N1 virus [36] and 26E in H9N2 AIV [42]. Mechanistically, PA-X selectively targets RNA Pol II-transcribed host mRNAs in the nucleus [14], and this activity is tightly linked to host Pol II transcript splicing [21]. Meanwhile, accumulating studies have also demonstrated the crucial role of PA-X in regulating the viral virulence of different IAV strains [8,10,23–31]. While the role of PA-X in immune evasion and viral virulence is well established [14,18,21,43–45], no data clearly defined the connection of PA-X host shutoff activity with viral fitness of IAV in various animal models.

In this study, on one hand, we aimed to pinpoint the crucial host shutoff sites in the PA-X protein of the highly pathogenic H7N9 virus. On the other hand, we were curious about whether this site/these sites affect viral fitness in mammals and birds. As a result, we mapped the site PA-X 100 V that contributed to the host shutoff activity of H7N9 and H5N1 virus in human 293T cells, which is quite consistent with previous findings that the N-terminal of PA-X contributes to the host shutoff activity (Figure 2) [9,19,36,37]. Structure modelling revealed that PA-X 100 V located around the nuclease active site and extended towards the endonuclease active site, suggesting its potential structural link with endonuclease activity (Figures 9a-d). Furthermore, the site PA 100 V was close to the PB2-Lid domain and PB1-terminal extension domain (Figures 9e,f), which may correlate with PA-X 100 V’s role in regulating viral replication in mice and chickens (Figure 3–6). Subsequently, we discovered that the host shutoff site PA-X 100 V enhanced viral fitness in mice (Figure 3,4) while it decreased this aspect in chickens (Figure 5,6). Further mechanistic studies revealed that PA-X 100 V substantially increases viral polymerase activity and viral replication. Global transcriptomic analysis demonstrated that PA-X 100 V markedly involved in PA-X-mediated degradation of host genes in human 293T cells (Figure 10–13, Figures S1, S2, and S5) but not in DF-1 cells (Figure 10, Figures S3, S4, S7, and S8). More specifically, in 293T cells, PA-X 100 V selectively targets cellular mRNAs involved in electron transport chain and the inflammatory response signalling pathway (Figure 13, 14, Excel S1). By contrast, in DF-1 cells, PA-X 100 V showed no obvious preference of degrading the specific class of genes (Figure 10, Figure S4, Figure S8).

As the energy power plants of cells, mitochondria generate ATP *via* the respiratory electron transport chain. Here, we provided some proof that PA-X 100 V restrained respiratory electron transport chain-related genes in 293T cells (Figure 13) and MDCK cells (Figure 14). Usually, viral replication is extremely dependent on energetic processes [46]. Hence, it is highly possible that the preference of PA-X 100 V to regulate electron transport chain and inflammatory-related response in mammalian cells may provide an optimum cellular environment for efficient viral fitness in mice. However, the underlying mechanism that allows PA-X 100 V to selectively target these host RNAs in 293T cells for destruction still needs to be further determined. One possible explanation is that the connection of PA-X 100 V on NS1 translation may affect the overall viral host shutoff ability and contribute to the degradation of these host genes [47]. In addition, PA-X usurps RNA splicing to selectively target host Pol II-transcribed RNAs for destruction by interacting with host cellular RNA processing proteins [21]. At this stage, it is unclear whether the single PA-X A100V mutation alters the interaction with these proteins and results in the observed target preference for energy metabolism and inflammatory response-related genes. However, it is also possible that PA-X 100 V may cause selective downregulation of host mRNAs by other novel mechanistic determinants, not by the established model that discriminates Pol II-dependent RNA biogenesis in the nucleus [14]. Nuclear localization of PA-X is tightly linked to its ability to target nuclear mRNA pools [14]. However, we found that PA-X 100 V had no significant effect on PA accumulation in the nucleus (Figure 8). Therefore, a major challenge for our future studies will be to disentangle the highly interconnected steps of energy metabolism to better understand the function of PA-X 100 V.

Previous studies demonstrated that a higher inflammatory response is correlated with enhanced virulence *in vivo* during infection with PA-X-deficient recombinant viruses [10,23,24,33]. By contrast, in our study, compared with PA-X 100 A, although PA-X 100 V significantly enhanced viral replication and virulence in mice, it had no obvious effect on cytokine expression in mouse lung (Figure 4). We surmised that this phenomenon actually reflected the host shutoff ability of PA-X 100 V in mice because the rising cytokines’ response that accompanied the high replication of the mutant GD15-A100 V virus may have been restrained by PA-X 100 V (Figure 4c). Also, there can be other explanations. One possible explanation is that other viral proteins, such as the NS1 [13,17,18,48] and PB1-F2 proteins [31], may act synergistically with the PA-X protein to fine-tune the overall host cytokine response. Another possibility is that PA-X 100 V-mediated host shutoff activity may be overwhelmed by a dominant PA-specific replication effect *in vivo* [42]. Lastly, the host shutoff feature of H7N9 PA-X may be quite different from that of other subtypes. Consistently, our previous study showed that PA-X deficiency had no significant effect on viral virulence and cytokine response in H7N9 virus-infected mice [49]. Distinctive findings were also obtained in studies on H9N2 AIV [25] and H3N8 and H3N2 canine influenza viruses [35]. For example, the absence of PA-X in H9N2 virus attenuated viral pathogenicity and dampened the pro-inflammatory cytokine and chemokine response in mice [25]. Therefore, our study added further supportive evidence that the PA-X shutoff activity on the host innate immune response varies among different influenza virus strains *in vivo*.

Adaptive mutations are an important mechanism for cross-species transmission of AIV. For example, K615N and T97I mutations of the H7N9 PA gene increase viral replication and pathogenicity in mammals [50,51], and A70V and P224S mutations of the H1N1 PA gene enhance viral fitness in mice [52]. Our results clearly showed that the PA-X A100 V mutation markedly increased viral replication and virulence of H7N9 AIV in mice but decreased viral fitness in chickens (Figure 3–5), suggesting an important role of this site in host adaptation of H7N9 AIV in mammals. Interestingly, several studies have demonstrated the important role of PA/PA-X in regulating viral characteristics [41,53,54]. Nogales *et al*. revealed that the recent 2009 pH1N1 viruses containing amino acid changes in NS1 protein (E55K, L90I, I123V, E125D, K131E, and N205S) and amino acid changes in PA-X protein (V100I, N204S, R221Q, and L229S) had increased NS1-mediated inhibition of host gene expression and decreased PA-X-mediated shutoff [41]. In addition, the recombinant pH1N1 virus containing PA, PA-X, and NS1 genes from currently circulating viruses is fitter in mice than the original ancestor pH1N1 virus. However, this study did not pinpoint the crucial amino acids(s) that affect the host shutoff of PA-X protein. Meanwhile, Yang and Meng *et al*. both revealed that PA V100I mutation increased the pathogenicity of Eurasian avian-like H1N1 swine influenza in mammals *via* enhancing the endonuclease cleavage activity and viral RNA-binding ability of the PA protein [53,54]. Lutz *et al*. showed that PA V100I mutation led to greater synthesis of viral proteins, most notably HA, NP, M1, and NS1 [47]. Considering the important role of NS1 in host shutoff and innate immunity, we surmised that the host shutoff activity of PA A100V may have a connection with its potential role in regulating the translation of NS1 protein. In addition, the fate of viral mRNAs and proteins during host shutoff has been a topic of intense study, as the expectation is that viral products should be spared from RNA degradation. Therefore, our future studies will also determine whether PA-X A100 V-mediated host shutoff influences the efficiency of viral gene expression, especially the NS1 and PB1-F2 proteins.

A previous study demonstrated that amino acid residues P28 and S65 contributed to the shutoff activity of PA-X in multiple subtypes of influenza A virus [36]. In this study, PA-X 100 V not only enhanced host shutoff in the H7N9 subtype but also in the H5N1 subtype (Figure 2). Gene polymorphism analysis of H7N9 and H5N1 viruses showed that PA-X 100 V is more prevalent in the H5N1 subtype than in the H7N9 subtype (Figure 1). Interestingly, the predominance of 100 V is quite stable in both avian (Figure 1i) and human H5N1 viruses from 1959 to 2023 and accounts for 100% from 2011 to 2023 in human H5N1 viruses (Figure 1j). The biological and evolutionary reasons why H5N1 influenza viruses constantly carry 100 V are not actually known. Hence, further studies investigating the role of PA-X 100 V in regulating the viral fitness and pathogenicity of H5N1 viruses in mammals and birds may partially explain the prevalence of this site in H5N1 IAV. Interestingly, a recent study demonstrated that the current circulating H5N1 virus showed enhanced PA-X host shutoff activity compared with early circulating H5N1 viruses, and the crucial sites were mapped to V127I and T118I [40]. However, there are no direct data regarding the particular role of the single V127I mutation or T118I mutation in regulating viral replication and virulence. Here, we directly proved that the novel identified host shutoff site PA-X 100 V directly regulates the viral fitness of H7N9 IAV in both mice and chickens. In addition, we also surmised that the H7N9 virus needs to optimize the contribution of 100 V and other host shutoff sites to circumvent host responses for its optimum growth and to facilitate viral fitness in various hosts.

In this study, we found that PA-X 100 V enhanced PA-X host shutoff ability in 293T cells while decreasing this aspect in DF-1 cells (Figure 5, 10). Meanwhile, PA-X 100 V exerts a contrary effect in regulating viral virulence of the H7N9 influenza virus in mice and chickens (Figure 3–5). For this, we supposed that the PA-X 100 V mediated-vial phenotypes can track with its host shutoff activity. However, although the whole PA-X protein regulates host shutoff activity, we also found that loss of PA-X expression had no obvious effect on viral virulence in mice. We supposed that the divergence of PA-X and PA-X 100 V in regulating the overall host response and other potential host factors may together contribute to their phenotype in mice. In addition, we also surmised that our findings in mice with the A100 V virus may have a different explanation than simply regulating the known function of PA-X. For example, two independent studies confirmed that PA V100I increases viral mRNA transcription by enhancing endonuclease cleavage activity of the PA protein and further contributes to the increase of pathogenicity and transmissibility of Eurasian avian-like H1N1 swine influenza virus in mice and ferrets [53,54]. Therefore, we speculated that the endonuclease activity mediated by PA-X 100 V may be one of the factors influencing the viral phenotype and host shutoff activity. We also noted that although GD15-FS and the GD15-FS-A100 V mutant virus induced an obvious lung injury on days 3 and 5 post infection, no mice was dead in these groups. One possible explanation was that lung injury may disappear at later infection times in these groups. Therefore, further studies are needed to monitor lung injury at later times, such as at days 7, 9, 11, and 13 post-infection. If any, the precise mechanism of restoring lung damage mediated by loss of PA-X expression at later infection times still needs to be determined. We surmised that the altered overall innate immune response mediated by down-regulation of PA-X expression may contribute to the alleviation of lung damage at a later infection time.

As for viral polymerase activity, PA-X-A100 V not only enhanced viral polymerase activity in 293T cells but also in DF-1 cells (Figure 7), suggesting that polymerase activity may not be the factor that determining the inverse role of PA-X 100 V in regulating viral fitness of H7N9 virus in mice and chickens. Moreover, consistent with the polymerase activity results in DF-1 cells (Figure 7), PA-X 100 V also increased viral replication in DF-1 cells and CEF cells (Figure 5). However, to our great surprise, PA-X 100 V did not enhance viral replication and virulence in chickens. We surmised that the intricate role of PA-X 100 V in regulating host shutoff in chickens may partially account for this phenomenon. In addition, the great discrepancy of the innate immune response between mice and chickens may be another factor. In addition, considering the important roles of 100 V in host adaptation in mammals and chickens, further studies elucidating the precise mechanism of this action will accelerate our understanding of the complexity of IAV pathogenesis. For example, determination of the particular functional connection between 100 V-targeted mRNA and viral pathogenicity, especially those genes involved in regulating the electron transport chain and mitochondrial membrane function. Besides, for the inverse role of PA-X 100 V in regulating host shutoff activity of PA-X in 293T and DF-1 cells, we speculated that the expression of the highly active PA-X endorsed by PA-X 100 V in mammalian 293T cells might not be optimal for H7N9 virus in avian DF-1 cells due to the discrepancy in their host cellular machinery.

In summary, the present study identified PA-X 100 V as the crucial site that contributed to the PA-X host shutoff ability and viral fitness of H7N9 virus. Notably, the host shutoff site 100 V exerted contrary roles in manipulating viral replication and virulence of H7N9 virus in mice and chickens. For enhanced virulence in mammals, mechanistically, PA-X 100 V markedly increased viral replication and polymerase activity in mammalian cells. Moreover, PA-X 100 V played an important role in reorganizing the global host response in 293T cells, which was characterized by its profound effect on blunting cellular energy metabolism and the inflammatory response. Thus, our findings highlight the important role of PA-X host shutoff activity in the pathogenesis of H7N9 AIV and provide important implications for understanding the pathogenesis of IAV. However, additional studies are required to further understand the precise mechanism for the enhanced shutoff ability mediated by PA-X 100 V and its subsequent contribution to regulating viral pathogenesis.

## Materials and methods

### Ethics statement

This study was carried out in strict accordance with the recommendations in the Guide for the Care and Use of Laboratory Animals of the Ministry of Science and Technology of the People’s Republic of China. The protocols for animal experiments were approved by the Jiangsu Administrative Committee for Laboratory Animals (ethical approval number: SYXK-SU-2021–0027 for chickens and SYXK-SU-2021–0026 for mice) and complied with the guidelines of Jiangsu laboratory animal welfare and ethics of Jiangsu Administrative Committee of Laboratory Animals. All experiments involving live viruses and animals were housed in negative-pressure isolators with HEPA filters in a biosafety level 3 (BSL3) animal facilities in accordance with the institutional biosafety manual.

### Viruses and cells

A/chicken/Guangdong/GD15/2017 H7N9 (GD15) virus was isolated in live poultry markets in 2017 [55] and propagated in 9-day-old specific-pathogen-free (SPF) embryonated chicken eggs. The reverse genetic-derived GD15 virus and the corresponding PA-X- deficient virus (GD15-FS), which carried five mutations in the frame shifting motif in the PA-X gene were generated previously [56]. The mutant virus GD15-A100V harbouring A100V mutation in the GD15 PA gene and the mutant virus GD15-FS-A100V containing A100V mutation in PA gene of the PA-X- deficient virus (GD15-FS) were generated as previously described [8]. All the rescued viruses were sequenced to verify the presence of the introduced mutations and the absence of unwanted mutations. Canine MDCK, human 293T, human A549 cells, avian DF-1 cells, and CEF cells were cultured in Dulbecco’s modified Eagle’s medium (DMEM) (Thermo Fisher Scientific, Carlsbad, CA, USA) supplemented with 10% foetal bovine serum (FCS) (Thermo Fisher Scientific) and were incubated in a 37°C, 6% CO_2_ incubator.

### Sequence analysis

The PA-X polymorphism of the IAV was analysed based on the data in GISAID (https://platform.epicov.org/epi3/frontend). All available PA-X gene sequences (until June 2024) of the avian- and human-origin H5N1, H5N6, H5N8, H9N2, and H7N9 subtype viruses were analysed. Sequences of PA-X were aligned using MUSCLE (multiple sequence comparison by Log-Expectation).

### Evaluation of the host shutoff activity of the PA-X protein

To pinpoint the critical residue(s) that contribute to the host shutoff activity of PA-X, 293T cells and DF-1 cells (24-well plate, 2 × 10^5.0^ cells/well, in triplicate) were cotransfected with 2 μg of the pires-hrGFP-1a plasmid that encodes the GFP protein together with 50 ng of the parental PA-X plasmid (p-GD15-PA-X), the PA-X mutant plasmids, or the empty vector pcDNA3.1-Flag using the PolyFect transfection reagent (QIAGEN, GmBH, Germany). The PA-X mutant plasmids were constructed by site-directed mutagenesis (TransGen, Beijing, China) based on the parental p-GD15-PA-X plasmid (H7N9 PA-X) or p-CK10-PA-X (H5N1 PA-X). At 48 h p.t., GFP expression was evaluated under a fluorescence microscope and its intensity was calculated by Image J.

To determine the impact of PA-X 100 V on the global host gene expression mediated by the PA-X protein, 293T cells or DF-1 cells were transfected with either the pcDNA3.1-Flag vehicle or the plasmids expressing the wt or mutant PA-X gene together with *Renilla* luciferase plasmid (QIAGEN). At 24 h p.t., *Renilla* luciferase production was measured using *Renilla*-Glo luciferase assay system (Promega, Madison, WI, USA).

### Western blotting

The corresponding samples in host shutoff assay were further analysed by Western blotting. The Flag antibody was used as the primary antibody (Beyotime Biotechnology, Nantong, China). As for the determination of the PA-X level in 293T cells transfected with different PA plasmids, the PA-X mouse monoclonal antibody (prepared in our lab) was used. The blots were washed three times for 10 min each in TBST buffer and incubated for 1 h at room temperature with horseradish peroxidase-conjugated anti-mouse or -rabbit IgG (Sigma, St. Louis, MO). The mouse monoclonal antibody β-actin or β-tubulin was used as an internal standard (Abcam, Cambridge, United Kingdom). The relative levels of the PA-X protein to control expression were determined by densitometry using Bandscan 5.0 software. Data shown are representative images of three independent experiments.

### Determination of virus replication in vitro

To investigate the effect of the host shutoff site 100 V on viral replication of the H7N9 virus in different cells, canine MDCK, human A549, human 293T cells, avian DF-1 cells, and CEF cells were inoculated at a MOI of 0.01. Supernatants collected at 12, 24, 36, 48, and 60 h p.i. were then subjected to titration by inoculating MDCK cells in 96-well plates. The 50% tissue culture infectious dose (TCID_50_) titres were used for calculating as viral titres based on the Reed and Muench method [57].

### Polymerase reconstitution assay

To determine the effect of A100V mutation on viral polymerase activity, 293T cells or DF-1 cells were transfected with 200 ng of luciferase reporter plasmid (p-Luci), 20 ng internal control *Renilla* plasmid pRL-TK, 200 ng of pcDNA3.1+ plasmid constructs expressing the polymerase subunits PB2, PB1, NP and parental PA (p-GD15), or the mutant PA (p-GD15-A100 V or p-GD15-FS or p-GD15-FS-A100V), respectively. At 24 h p.t., cell lysates were prepared using Dual-Luciferase Reporter Assay System (Promega), and the reporter gene expression was determined by measuring luminescence using a GloMax 96 microplate luminometer (Promega). Samples were measured in independent biological triplicate experiments. For determining the expression of the RNP complex components, 293T cells or DF-1 cells mentioned above were collected, and the specific antibodies for PB1, PB2, PA and NP were used as the primary antibodies (GeneTex, Texas, USA), respectively.

### Mouse experiments

To evaluate the role of PA-X A100V mutation in the pathogenicity H7N9 virus in mice, a total of 65 healthy 6-week-old female BALB/c mice (Yangzhou University Laboratory Animal Center, Yangzhou, China) were randomly grouped into 13 groups (5 mice/group), GD15, GD15-A100 V, GD15-FS, GD15-FS-A100 V, and PBS (mock control), respectively. For the virus group, each contains three sub-group, which inoculated a dose of 10^4.0^, 10^5.0^, and 10^6.0^ EID_50_ of each virus, respectively. Under mixed anaesthesia (medetomidine-butorphanol-midazolam), the mice were then intranasally (i.n.) inoculated with serial dilutions (10^4.0^ ~ 10^6.0^ EID_50_ in 50 μL PBS) of the indicated viruses (each dilution of virus infected 5 mice) for determining the MLD_50_ of the viruses. An uninfected control group was anesthetized and intranasally inoculated with 50 μL of PBS. To minimize the potential confounders, two persons who were not aware of this experiment were designed to record the daily weight, mortality, and morbidity of the infected mice. Animals were monitored daily for mortality over a period of 14 days as previously described [58]. Animals showing severe disease signs or lose above 25% of their initial weight were euthanized and recorded as having died on the following day [59]. MLD_50_ was calculated using the Reed and Muench method [57].

To investigate virus replication *in vivo*, a total of 30 healthy 6-week-old female BALB/c mice (Yangzhou University Laboratory Animal Center, Yangzhou, China) were randomly grouped into 5 groups (6 mice/group), including GD15, GD15-A100V, GD15-FS, GD15-FS-A100V, and PBS mock control group. Under mixed anaesthesia (medetomidine–butorphanol–midazolam), the mice were then i.n. infected with 10^6.0^ EID_50_ in 50 μL PBS of the parental and mutant viruses. In addition, for comparison, the PBS group was anesthetized and intranasally inoculated with 50 μL of PBS. On day 3 and 5 p.i., three mice of each group were euthanized, and the heart, liver, spleen, lung, kidney, and brain were collected for virus titration in chicken eggs. In addition, another group of the collected lung tissues was used for determination of cytokine gene expression and histopathological analysis. The data analyses were performed using the independent-samples *t* test based on SPSS statistics software and were shown as the mean ± standard deviation (SD).

### Chicken experiments

To evaluate the role of A100 V mutation in the pathogenicity of H7N9 virus in chickens, a total of 25 healthy 5-week-old Specific Pathogen Free (SPF) chickens (Beijing Boehringer Ingelheim Viton Biotechnology Co., Ltd, Jiangsu, China) were randomly grouped into 5 groups (5 chickens/group), namely, GD15, GD15-A100V, GD15-FS, GD15-FS-A100V, and the PBS mock control. The chickens were then i.n. inoculated with (10^6.0^ EID_50_ in 100 μL 1× PBS) of the specified viruses. In addition, for comparison, the PBS group was i.n. inoculated with 100 μL of 1× PBS. Animals were monitored daily for mortality over a period of 14 days as previously described [58]. To minimize the potential confounders, two people who were not aware of this experiment were designed to record the daily weight, mortality, and morbidity of the infected chickens. Animals showing severe disease signs or lose above 25% of their initial weight were euthanized and recorded as having died on the following day [59].

To compare virus replication, a total of 30 healthy SPF 5-week-old chickens (Beijing Boehringer Ingelheim Viton Biotechnology Co., Ltd., Jiangsu, China) were randomly grouped into 5 groups (6 chickens/group), namely, GD15, GD15-A100V, GD15-FS, GD15-FS-A100V, and the PBS mock control. The chickens were then i.n. inoculated with (10^6.0^ EID_50_ in 100 μL 1× PBS) specified viruses. The group of PBS was i.n. inoculated with 100 μL of 1× PBS as mock control. At day 1 and 3 p.i., three birds per group were euthanized and the organs (heart, spleen, brain, and lung) were collected and homogenized by adding 900 µl of double antibiotic PBS per 0.3 g of tissue. Then, the homogenate was freeze–thawed three times at −80°C and 37°C and then centrifuged at 5000 rpm at 4°C, and the supernatant was inoculated into MDCK cells for the TCID_50_ assay. In addition, another group of the collected lung tissues was used for determination of cytokine gene expression and histopathological analysis. The data analyses were performed using the independent-samples *t* test based on the SPSS statistics software and were shown as the mean ± standard deviation (SD).

### Histopathological examination for mouse and chickens

Histopathological examination was performed as previously described [60]. Briefly, at days 1, 3, and 5 p.i. (mouse study) or day 1 and 3 p.i. (chicken study), the left lung was collected and fixed in 10% phosphate-buffered formalin and processed for paraffin embedding. Then, the tissue sections were stained with haematoxylin and eosin (H&E) and observed for histopathological changes. Images were captured with a Zeiss Axioplan 2IE epifluorescence microscope.

Lesion severity in mouse lung was scored according to the following standards: 0, no visible changes; 1, very mild lesions: alveolar capillaries were slightly dilated and congested; few oedema fluid was seen in the alveoli and mild alveolar haemorrhage; 2, mild lesions: alveolar capillaries were slightly dilated and congested; moderate oedema fluid was seen in the alveoli; mild haemorrhage in perivascular, bronchus, alveolar tube, alveolar sac, and alveolar; few lymphocytes infiltration in perivascular, bronchial, and alveoli; 3, moderate lesions: alveolar capillaries were slightly dilated and congested; severe oedema fluid was seen in the alveoli; haemorrhage in perivascular, bronchus, alveolar tube, alveolar sac, and alveolar; moderate epithelial cells, lymphocytes, and inflammatory cell infiltration in perivascular, bronchial, and alveoli; 4, severe lesions: alveolar capillaries were slightly dilated and congested; the alveoli were filled with oedema fluid; haemorrhage in perivascular, bronchus, alveolar tube, alveolar sac, and alveolar; a large number of epithelial cells, lymphocytes, and inflammatory cell infiltration in perivascular, bronchial, and alveoli.

The scoring criteria for chicken lung lesions were as follows: 0, no visible changes; 1, mild lesions, including minor congestion in parabronchus and/or pulmonary chamber; dilation of parabronchus and structure disappearance of the lung chamber; 2, moderate lesions, including moderate congestion or haemorrhage in parabronchus and/or pulmonary chamber; dilation in a large number of parabronchus and structure disappearance of the lung chamber; a few lymphocyte infiltration in blood vessels, parabronchus and/or pulmonary chamber; a small amount of eosinophilic material can be seen in the cavity; 3, severe lesions, including severe congestion or haemorrhage in bronchia, parabronchus, and/or pulmonary chamber; dilation in a large number of parabronchus and structure disappearance of the lung chamber; extensive lymphocyte infiltration around the parabronchus and/or pulmonary chambers; a number of eosinophilic materials can be seen in the cavity.

### Structural modeling of 100 V in PA-X protein and assembled polymerase complex

For structural modelling of 100 V in PA-X protein, the amino acid residue at 100 were mapped onto a ribbon diagram of the structure of the N-terminal region of PA (PDB accession of 2W69.2.A) by SWISS-MODEL (SWISS-MODEL.expasy.org) using the PyMOL software. Amino acid residues that form the endonuclease active site were shown in magenta. To understand the information about the position of PA-X 100 V in the assembled polymerase complex, the crystal structures of the polymerase were predicted using SWISS-MODEL Homology Modelling (ProMod3 3.3.0). The PDB: 6evj.2 Cryo-EM structure of influenza RNA polymerase was served as the template. The global and per-residue model quality has been assessed using the QMEAN scoring function [61].

### RNA extraction and purification for RNA-seq

The 60–80% confluent 293T cells or DF-1 cells in a 12-well plate were either transfected or un-transfected with the 2 µg of each plasmid in triplicates. At 24 and 48 h p.t., total RNA was extracted from the cells using RNeasy mini kit (QIAGEN) following the manufacturer’s instructions. Agilent Bioanalyzer 4200 (Agilent Technologies, CA, USA) was used to analyse RNA Integrity (Beckman Coulter, Inc. Kraemer Boulevard Brea, CA, USA). RNase-Free DNase Set (QIAGEN) was used to purify the total RNA and the samples with a ratio of OD_260_/_280_ of approximately 2.0 were used in the following experiments.

### Library construction for RNA-seq and sequencing procedures

*Paired-end* libraries were synthesized by using the TruSeq® RNA Sample Preparation Kit (Illumina, USA) based on the TruSeq® RNA Sample Preparation Guide. Briefly, the poly-A containing mRNA molecules were purified using poly-T oligo-attached magnetic beads. After purification, mRNA was fragmented into small pieces using divalent cations under 94°C for 8 min. The cleaved RNA fragments were copied into the first strand cDNA using reverse transcriptase and random primers. This was followed by the second strand cDNA synthesis using DNA Polymerase I and RNase H. These cDNA fragments then go through an end repair process, the addition of a single “A” base, and then ligation of the adapters. The products were then purified and enriched with PCR to create the final cDNA library. Purified libraries were quantified by Qubit® 2.0 Fluorometer (ThermoFisher Scientific) and validated by Agilent 4200 bioanalyzer (Agilent Technologies) to confirm the inserted size and calculate the mole concentration. Clusters were generated by cBot with the library diluted to 10 pM and then were sequenced on the Illumina NovaSeq (Illumina). The library construction and sequencing were performed at Shanghai Silver Crown Biomedical Technology Co., Ltd.

### Data analysis for gene expression

Sequencing raw reads were preprocessed by filtering out rRNA reads, sequencing adapters, short-fragment reads, and other low-quality reads. Tophat v2.1.0 [62] were used to map the cleaned reads to the human h38 reference genome (for 293T cells) or avian GRCg7b.110 (for DF-1 cells) with two mismatches. After genome mapping, Cufflinks v2.1.1 [63] was run with a reference annotation to generate FPKM values for known gene models. The SDE genes were identified using Cuffdiff [63]. The *p*-value significance threshold in multiple tests was set by the false discovery rate (*FDR*) [64]. The fold-changes were also estimated according to the FPKM in each sample. SDE genes were selected using following filter criteria: *p*-values < 0.05 and fold-change ≥ 2.

### Gene ontology and Kyoto encyclopedia of genes and genomes pathway analyses

The Database for Annotation, Visualization and Integrated Discovery (DAVID) (https://david.ncifcrf.gov/home.jsp) was used to analyze the SDE gene-associated Gene Ontology (GO) and Kyoto Encyclopedia of Genes and Genomes (KEGG) pathways. These data were log_2_-transformed and median-centred using the Adjust Data function of the *R* package plots. Hierarchical clustering using the *R* package average linkage was then performed, and DAVID assigned these genes to the relevant GO biological process and KEGG molecular pathways. Related and significant GO biological processes were identified by EASE score *p*-values of <0.01, and the significant KEGG molecular pathways were identified by the EASE score *p*-values of <0.05. Normally, higher enrichment and gene counts indicated more important pathways. Finally, tree visualization was performed using Java Tree view (Stanford University School of Medicine, Stanford, CA, USA).

### Expression profiles of the representative host genes

The expression of the representative genes screened by RNA-Seq in 293T cells, including CPZ, OAS1, CCL5, SOCS1, BCL2L11, and ITGAM, and those of the PA-X 100 V preferentially regulated genes were validated using qRT-PCR. Total RNA was isolated from the transfected-293T cells or virus infected MDCK cells (at a MOI of 2) using TRIzol reagent (ThermoFisher Scientific) and treated with DNase I (ThermoFisher Scientific). A total of 1 μg RNA per sample was reverse-transcribed into cDNA using 400 U RevertAid Premium reverse transcriptase (ThermoFisher Scientific) and 100 M random primers in the presence of RNase inhibitor (ThermoFisher Scientific) at 50 °C for 30 min. The qRT-PCR mixture contained cDNA, 200 nM of each primer, and 10 μL of 2 × SYBR green PCR master mix (TaKaRa, Shiga, Japan). The qRT-PCR was performed in triplicate using an ABI Prism 7900 system (Applied Bio systems, CA) with the following cycle profile: 1 cycle at 50°C for 2 min and 1 cycle at 95°C for 5 s followed by 40 cycles at 95°C for 5 s and 60°C for 31 s. For all reactions, one cycle for melting curve analysis was added to verify product specificity. The expression value for each gene relative to that of the *β*-actin was calculated using the threshold cycle 2^−ΔΔ*Ct*^ method.

### Measurement of PA and NP nuclear accumulation

Both indirect immunofluorescence assay (IFA) and Western blotting (WB) were used for determining the nuclear accumulation of the PA and NP proteins in MDCK cells. For IFA, MDCK cells were grown in 12-well plates and infected with the indicated viruses at a MOI of 2. At the indicated time points, the cells were fixed with PBS containing 4% paraformaldehyde for 20 min, saturated with PBS containing 0.5% Triton X-100 for 10 min and then blocked with 10% bovine serum albumin in PBS for 30 min. The cells were then incubated with rabbit anti-sera against PA (GeneTex) or monoclonal antibody against NP (GeneTex) at 37°C for 1 h. The cells were then washed three times with PBS and incubated at 37°C for 1 h with fluorescein isothiocyanate (FITC)-coupled donkey anti-rabbit or goat anti-mouse secondary antibodies (Beyotime Biotechnology). After washing for three times, the cells were then incubated with 4,’6-diamidino-2-phenylindole (DAPI) for 10 min. The stained samples were then observed under an LSM710 confocal microscope with Zen software (Carl Zeiss, Jena, Germany).

For WB, the MDCK cells were infected under the same conditions as for immunofluorescence analysis, followed by fractionation at 7 and 11 h p.i. Fractions were then analysed by immunoblotting for the distribution of the viral proteins, and the purity of the fractions was controlled by blotting for GAPDH and histone H3. More specifically, total cell lysates from fractionation experiments were heated at 80 °C for 5 min and subjected to 12% sodium dodecyl sulphate-polyacrylamide gel electrophoresis (SDS-PAGE) and were then transferred electrophoretically to a polyvinylidene difluoride (PVDF) membrane (Roche Diagnostics Corporation, Indianapolis, IN) using an electrophoresis system (Bio-Rad, Hercules, CA) and a Mini Trans-Blot electrophoretic transfer system (Bio-Rad). The membranes were blocked for 1 h at room temperature with Tris-buffered Saline-Tween (TBST) (20 mm Tris-HCl [pH 7.4], 137 mm NaCl, and 0.1% Tween 20) buffer containing 5% skim milk and were then incubated with primary monoclonal antibodies against PA or NP (Genetex), the anti-GAPDH antibody (Santa Cruz, CA, USA) or anti-histone H3 antibody (Beyotime) overnight at 4°C. The blots were washed three times for 10 min each time in TBST buffer and were incubated for 1 h at room temperature with horseradish peroxidase-conjugated anti-mouse or anti-rabbit IgG (Sigma, St. Louis, MO). Data presented are representative of the results of three independent experiments. Densitometry analysis was performed using the Image Studio Lite Software.

### Statistical analysis

Results were shown as the mean ± standard deviation (SD). Statistical analyses were performed using the independent-samples *t* test based on SPSS statistics software. All graphs were performed using Prism version 9 (GraphPad Software, San Diego, CA, USA). For all analysis, *p* < 0.05 (*), *p* < 0.01 (**), *p* < 0.001 (***) or *p* < 0.0001 (****) is considered as statistically significant between different groups.

## Supplementary Material

Excel S1 GO Enrichment analysis result of the downregulated SDE genes induced by p_GD15_A100V at 48 h pi.xls

FIG S1.tif

Table S1.docx

FIG S3.tif

The author checklist of ARRIVE guidelines.pdf

FIG S7.tif

FIG S6.tif

FIG S5.tif

FIG S2.tif

FIG S8.tif

FIG S4.tif

## Data Availability

All data generated or analysed during this study are deposited in Figshare, DOI: https://doi.org/10.6084/m9.figshare.26860537. All primary RNA-seq data have been deposited in the Gene Expression Omnibus (GEO) database (http://www.ncbi.nlm.nih.gov/geo/info/linking.html.) under the accession number GSE204790. The genomic sequences of GD15 virus are available in GenBank under the accession numbers (KY751288 for PB2, KY751256 for PB1, KY751223 for PA, KY751058 for HA, KY751157 for NP, KY751124 for NA, KY751091 for M and KY751190 for NS).

## Statement of adherence to ARRIVE guidelines

In this study, we followed the Animal Research: Reporting of In Vivo Experiments (ARRIVE) guidelines to ensure transparency and integrity in the design, execution, and reporting of our experiments. The ARRIVE guidelines are intended to improve the quality of scientific research, to ensure that experimental animal use is properly justified, and to promote the reliability and reproducibility of scientific discoveries. We have adhered to ARRIVE guidelines and uploaded a completed checklist as supplementary files.
