# Supplementary figures and images for: The PA-X host shutoff site 100 V exerts a contrary effect on viral fitness of the highly pathogenic H7N9 influenza A virus in mice and chickens
Source: Virulence. 2024 Dec 28;16(1):2445238. doi: 10.1080/21505594.2024.2445238 (PMC11702944; doi:10.1080/21505594.2024.2445238)

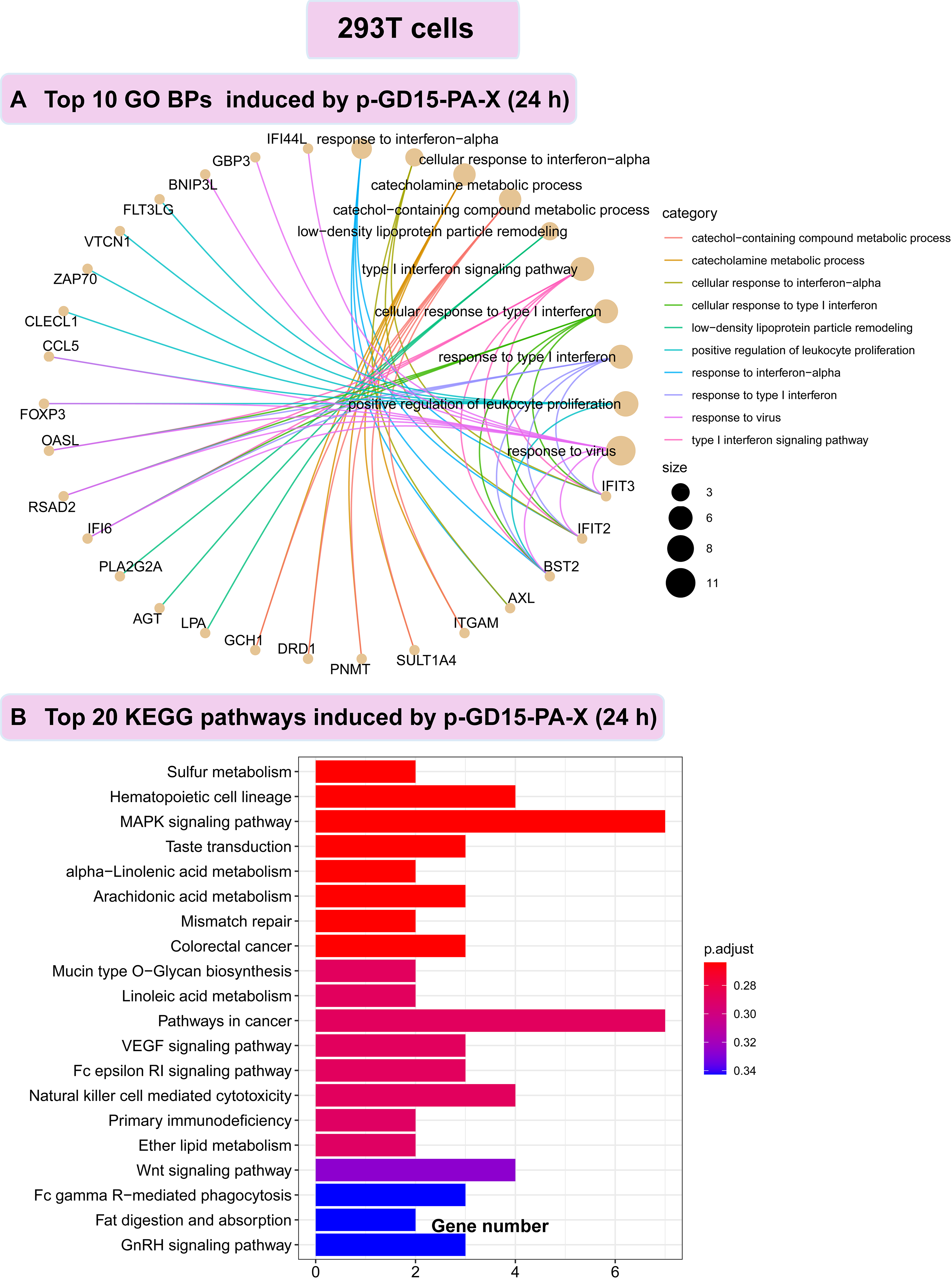

Supplement: FIG S1.tif [file KVIR_A_2445238_SM4564.tif]

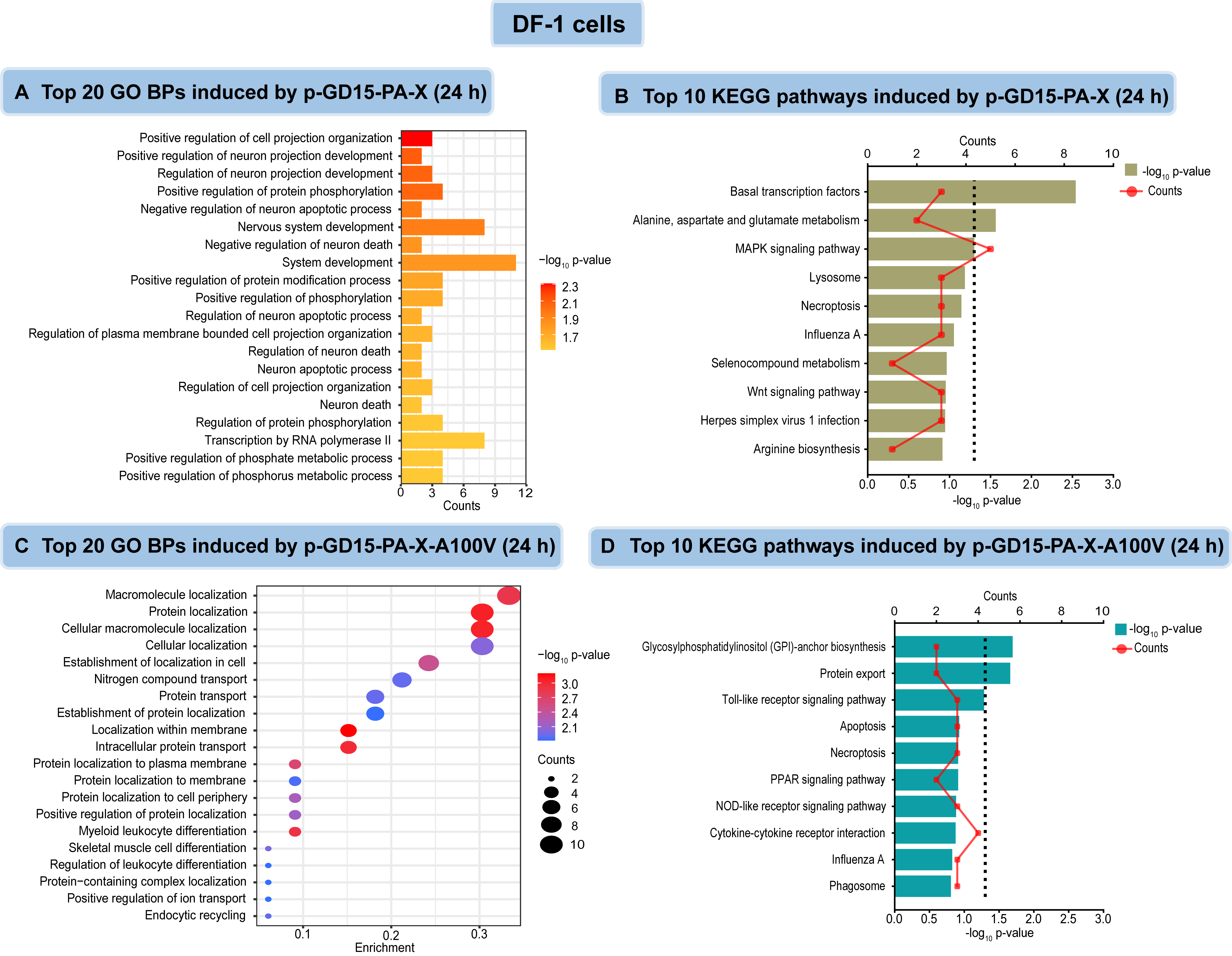

Supplement: FIG S3.tif [file KVIR_A_2445238_SM4562.tif]

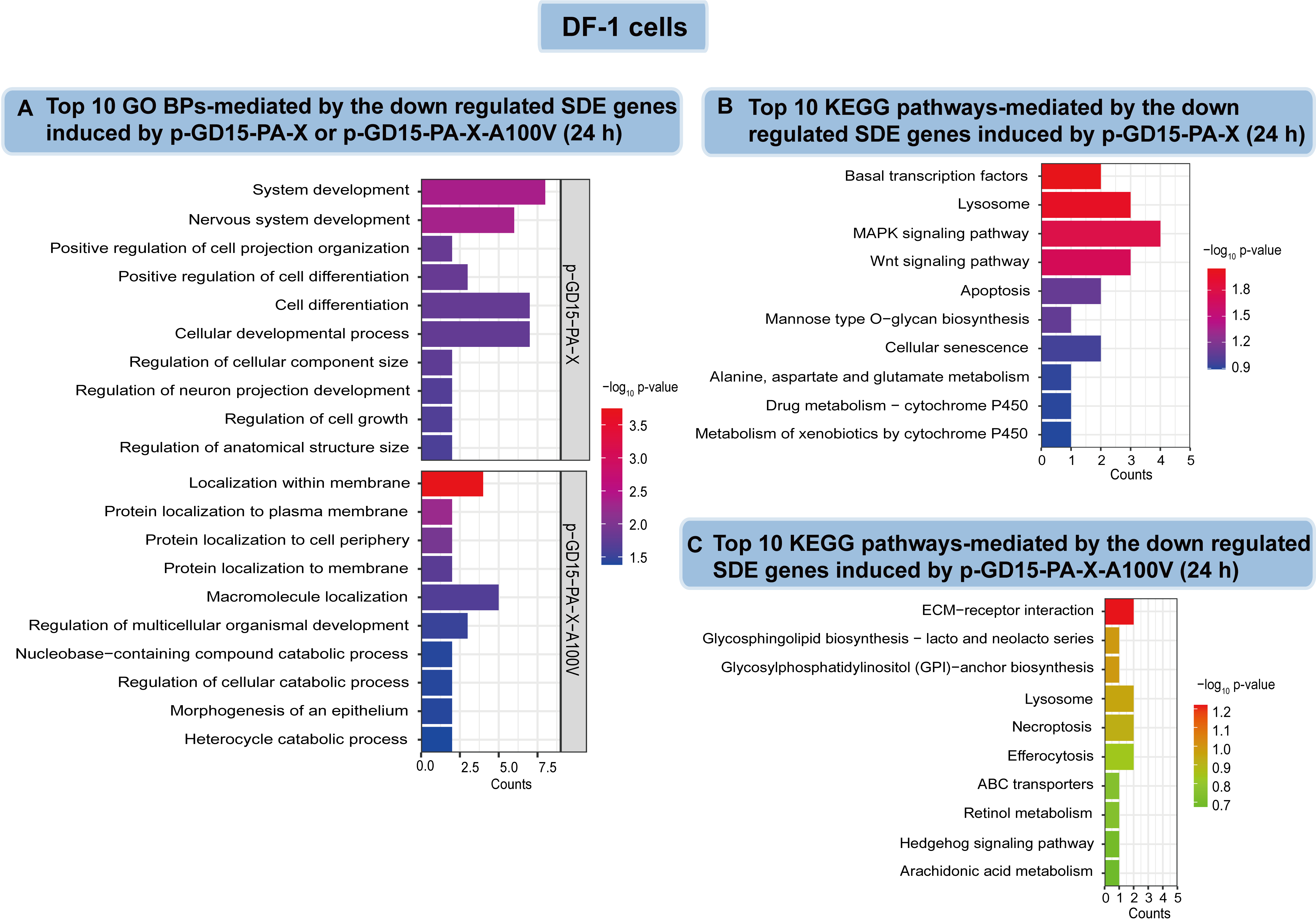

Supplement: FIG S7.tif [file KVIR_A_2445238_SM4560.tif]

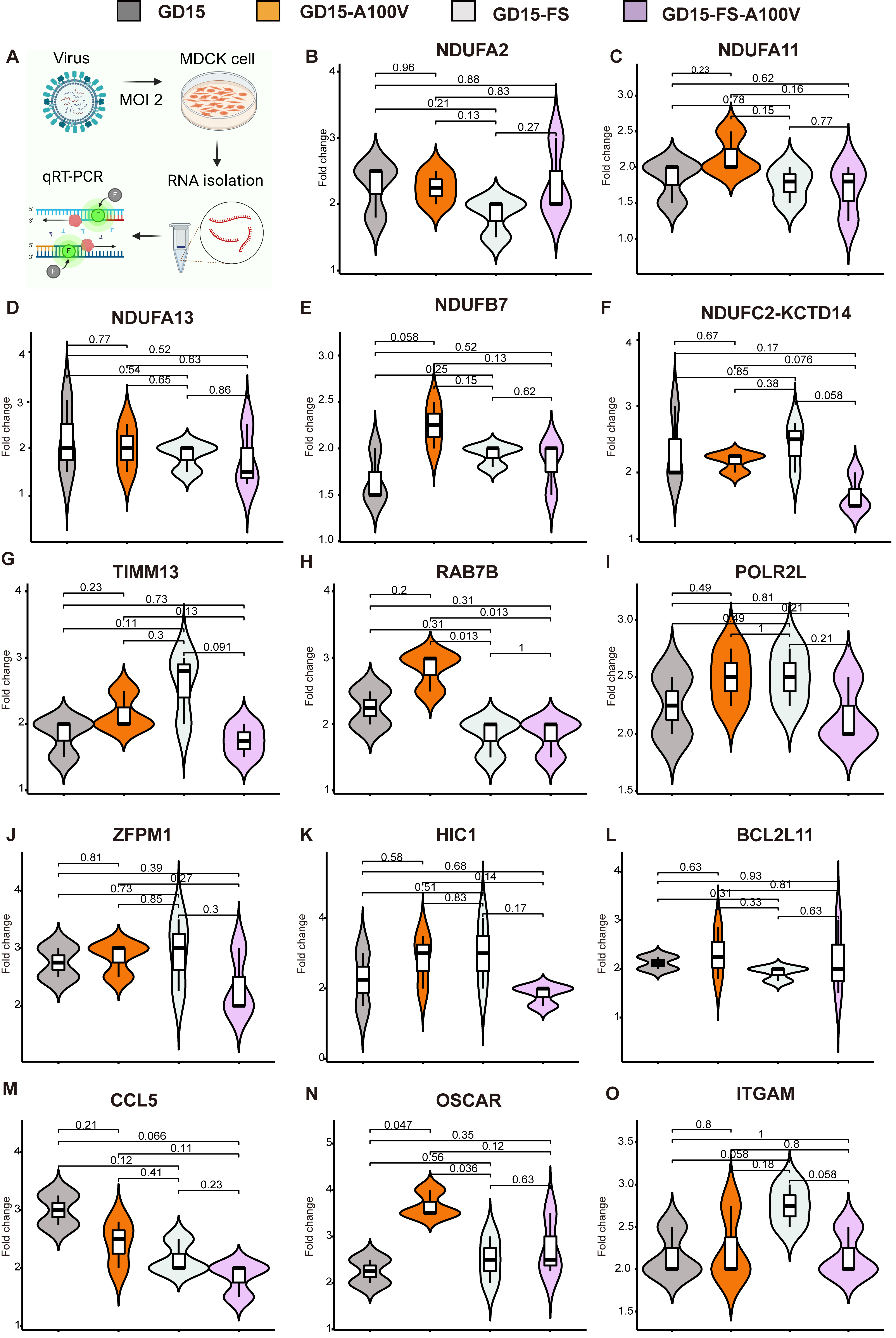

Supplement: FIG S6.tif [file KVIR_A_2445238_SM4559.tif]

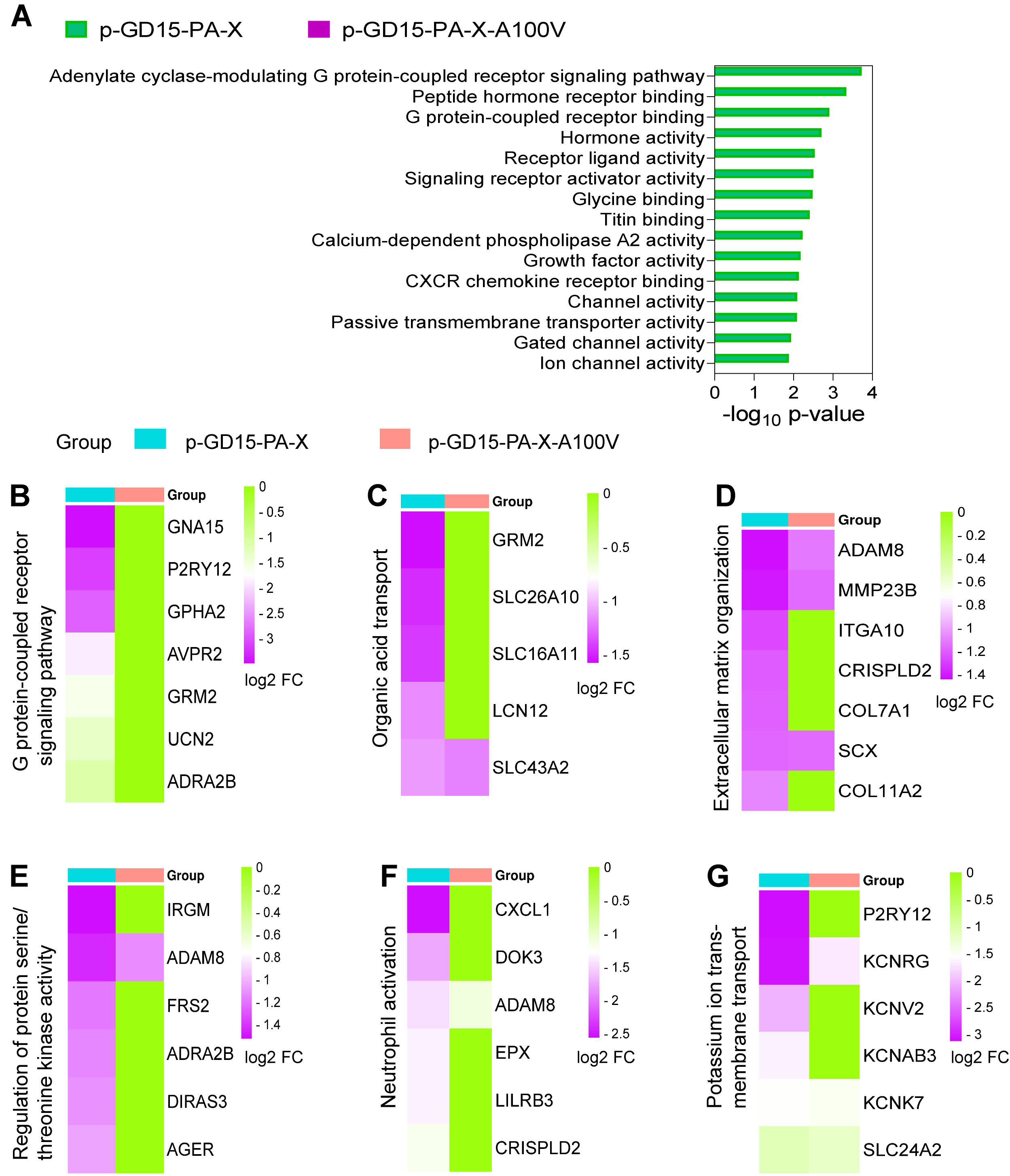

Supplement: FIG S5.tif [file KVIR_A_2445238_SM4558.tif]

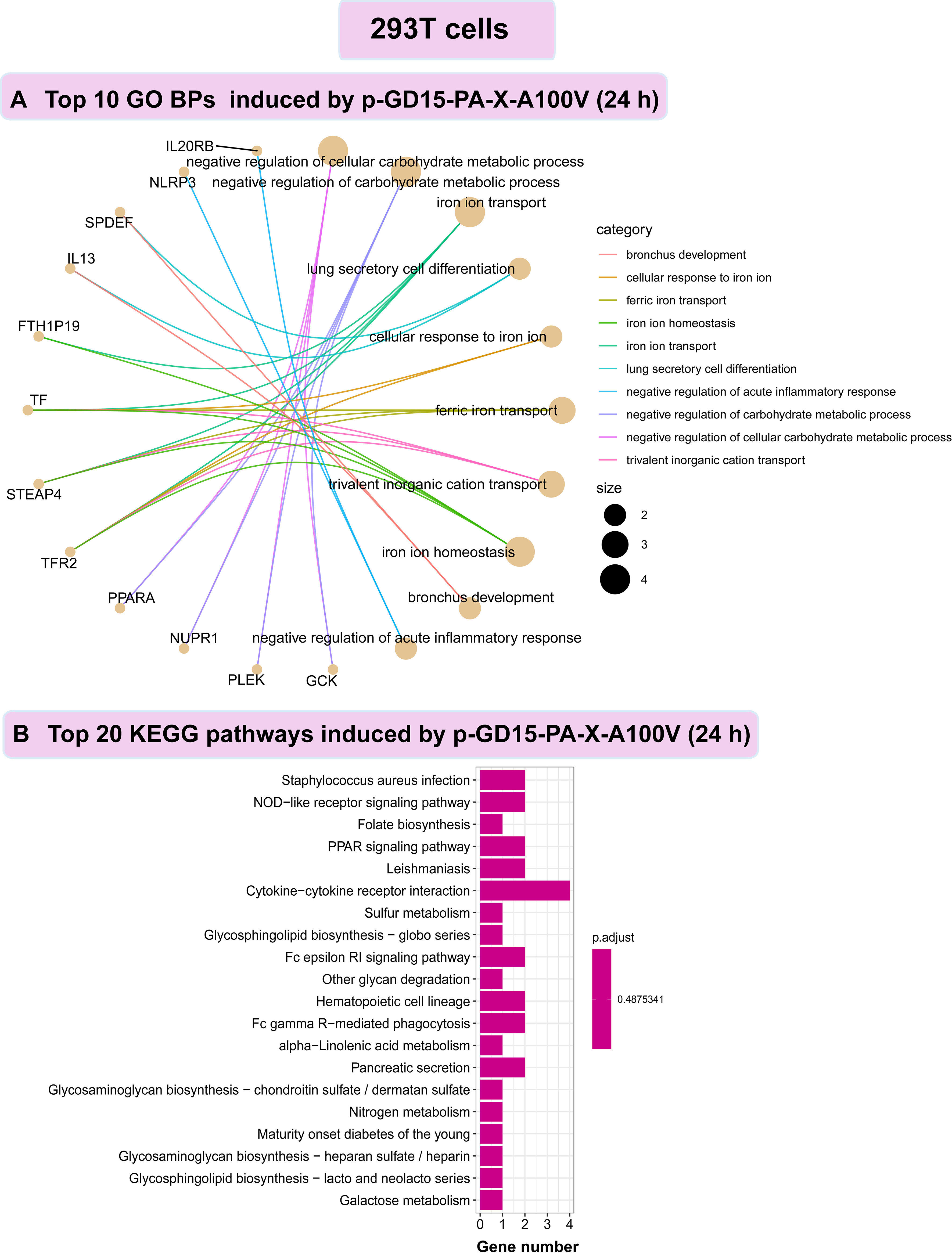

Supplement: FIG S2.tif [file KVIR_A_2445238_SM4557.tif]

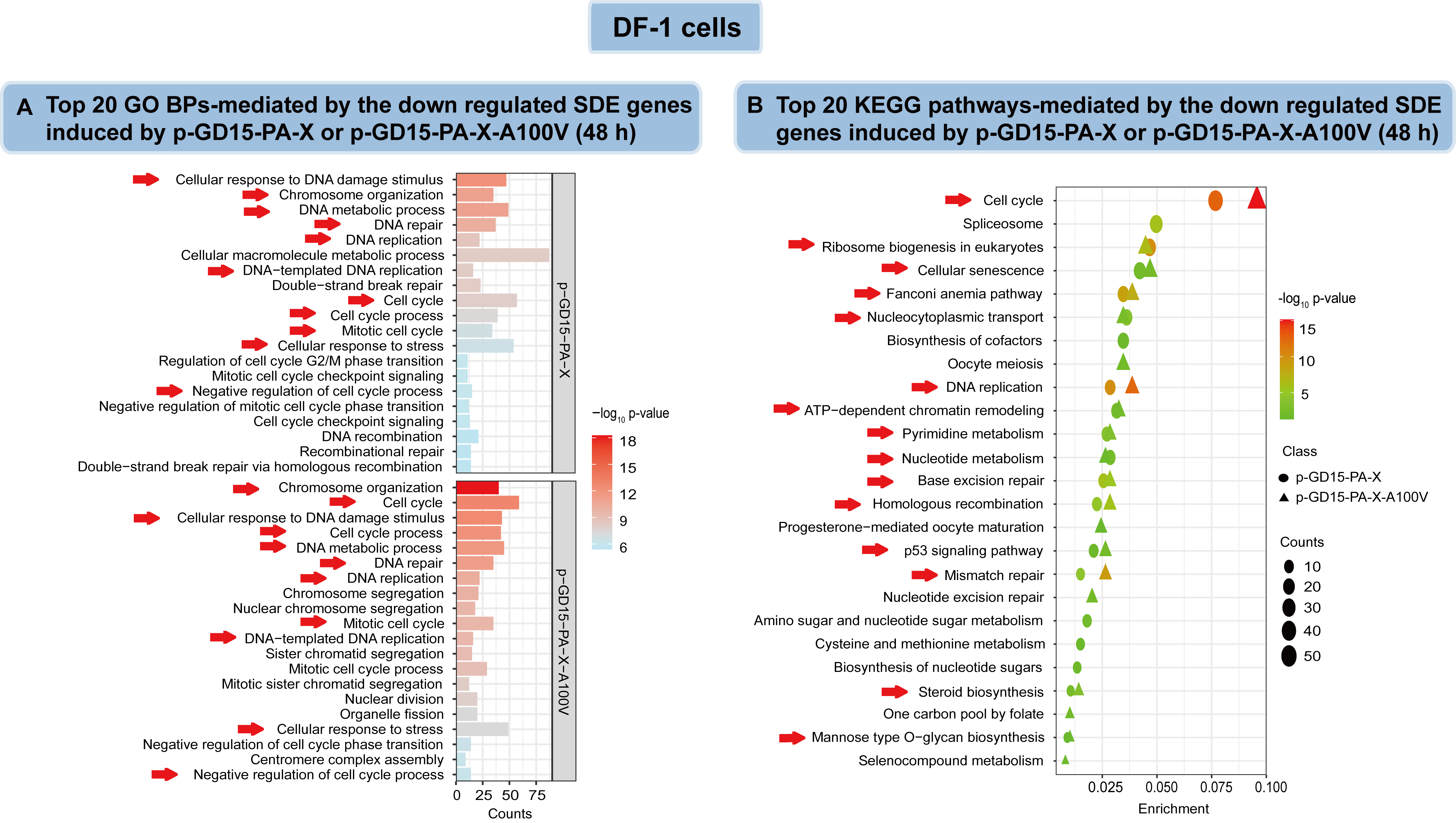

Supplement: FIG S8.tif [file KVIR_A_2445238_SM4556.tif]

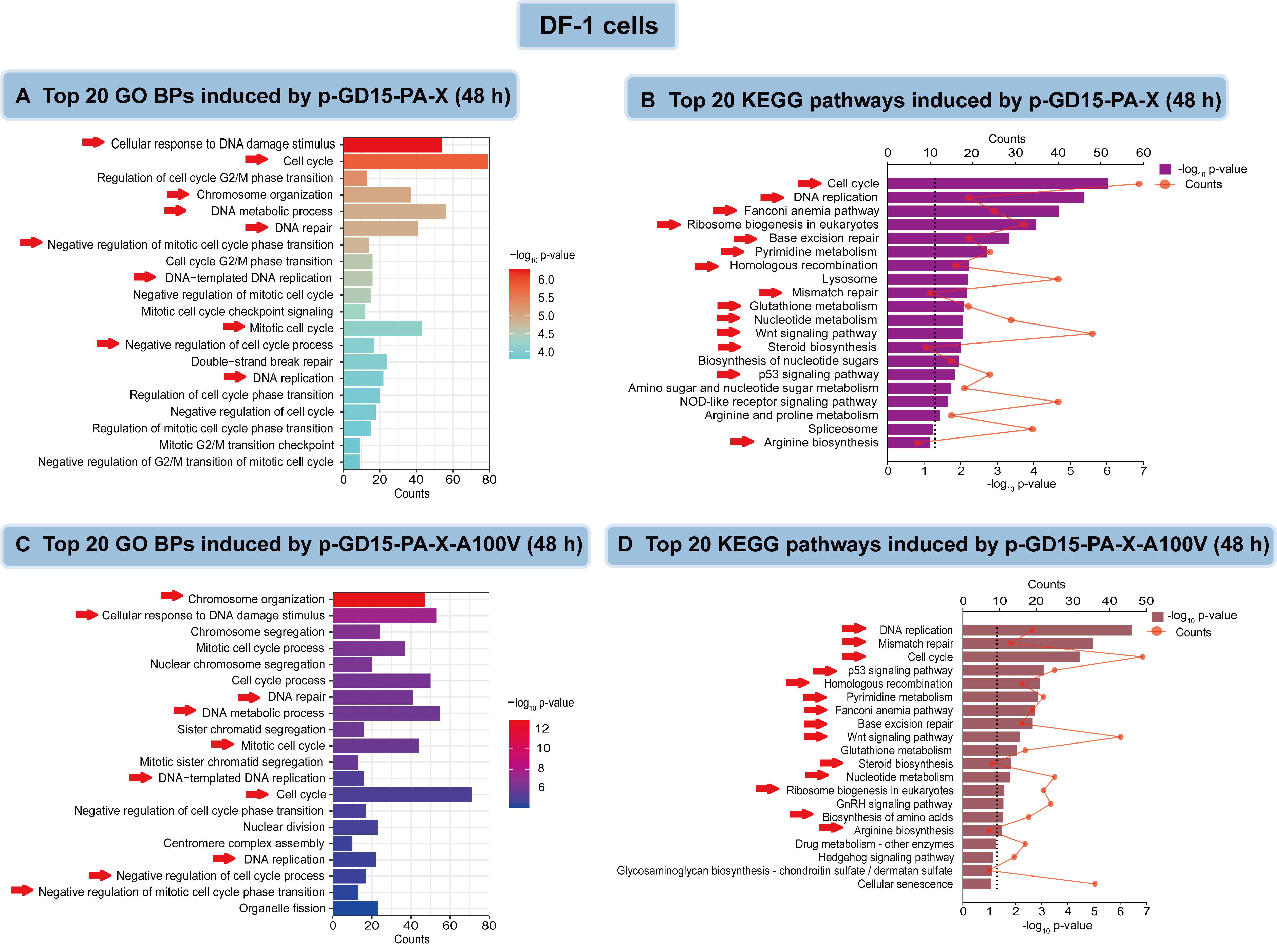

Supplement: FIG S4.tif [file KVIR_A_2445238_SM4555.tif]
