# Supplementary material for: The PA-X host shutoff site 100 V exerts a contrary effect on viral fitness of the highly pathogenic H7N9 influenza A virus in mice and chickens
Source: Virulence. 2024 Dec 28;16(1):2445238. doi: 10.1080/21505594.2024.2445238 (PMC11702944; doi:10.1080/21505594.2024.2445238)
Supplement: Table S1.docx [file KVIR_A_2445238_SM4563.docx]

**TABLE S1** **Specific residues/region that affect PA-X host shutoff activity**

| Region/Amino acid | Effect on shutoff activity | Available Information on Function | Reference |
| --- | --- | --- | --- |
| The helix α4 and the ﬂexible loop of amino acids 51 to 74 (Region) | Enhance | The flexible loop (residues 51 to 74) and helix α4 determine the difference in host shutoff activity between WSN and Cal virus | (1) |
| 195K/198K/199R (Region) | Enhance | Key residues for inducing host shutoff and nuclear localization | (2) |
| V100I, N204S, R221Q and L229S (Region) | Decrease | Prevalent in PA-X protein of recently circulating human pH1N1 viruses and account for better fitness in cultured cells and in mice | (3) |
| E2 (Amino acid) | Enhance | Required for PA-X shutoff activity in a NatB-dependent manner | (4) |
| E80, L106, P107, D108, E119, K134 (Amino acid) | Enhance | Residues required for nuclease activity of PA/PA-X | (5-8) |
| P28, S65 (Amino acid) | Enhance | Residues are important for viral polymerase activity | (9) |
| F4S, F9L, Y24S, D27G, C39Y, C45W, A87V, I94N, L106P/S184I, P107S, D108E, D108N, E119N, I120F, T123I, R124S, R125K, H146Y, E154K, E154A, D160Y, L163R, R168M, I171M (Amino acid) | Enhance | L106, P107, D108, E119 are required for nuclease activity of PA/PA-X; the side chains of Y24, C45, A87, I94, I120, L163, and I171 structurally support the formation of the nuclease active site | (8) |
| Amino acids 192 to 206, especially basic amino acids 195, 198, 199, 202, 203, 206 (Region) | Enhance | These six basic amino acids enabled a PA deletion mutant to suppress protein expression at a level comparable to that of wild-type PA-X | (10) |
| R195K (Amino acid) | Decrease | R195K mutation in the PA-X protein increases virulence and transmission of influenza A virus in mammalian hosts | (11) |
| L16P, N33S, K34E, E43K, F76L, P107S, L132P, H146Y, I147T, E153V, R170G, L187P, F191L (Amino acid); E101G/R125K, D111N/E196G（Region） | Decrease | Decrease shutoff activity; H146Y, L187P, and D111N/E196G also reduce viral polymerase activity | (12) |

**Reference**

1. Desmet EA, Bussey KA, Stone R, Takimoto T. 2013. Identification of the N-Terminal Domain of the Influenza Virus PA Responsible for the Suppression of Host Protein Synthesis. J Virol 87:3108–3118.

2. Hayashi T, Chaimayo C, McGuinness J, Takimoto T. 2016. Critical Role of the PA-X C-Terminal Domain of Influenza A Virus in Its Subcellular Localization and Shutoff Activity. J Virol 90:7131–7141.

3. Nogales A, Martinez-Sobrido L, Chiem K, Topham DJ, DeDiego ML. 2018. Functional Evolution of the 2009 Pandemic H1N1 Influenza Virus NS1 and PA in Humans. J Virol 92:e01206-18.

4. Oishi K, Yamayoshi S, Kozuka-Hata H, Oyama M, Kawaoka Y. 2018. N-Terminal Acetylation by NatB Is Required for the Shutoff Activity of Influenza A Virus PA-X. Cell Rep 24:851–860.

5. Hara K, Schmidt FI, Crow M, Brownlee GG. 2006. Amino acid residues in the N-terminal region of the PA subunit of influenza A virus RNA polymerase play a critical role in protein stability, endonuclease activity, cap binding, and virion RNA promoter binding. J Virol 80:7789–7798.

6. Dias A, Bouvier D, Crépin T, McCarthy AA, Hart DJ, Baudin F, Cusack S, Ruigrok RWH. 2009. The cap-snatching endonuclease of influenza virus polymerase resides in the PA subunit. Nature 458:914–918.

7. Yuan P, Bartlam M, Lou Z, Chen S, Zhou J, He X, Lv Z, Ge R, Li X, Deng T, Fodor E, Rao Z, Liu Y. 2009. Crystal structure of an avian influenza polymerase PA(N) reveals an endonuclease active site. Nature 458:909–913.

8. Oishi K, Yamayoshi S, Kawaoka Y. 2018. Identification of novel amino acid residues of influenza virus PA-X that are important for PA-X shutoff activity by using yeast. Virology 516:71–75.

9. Oishi K, Yamayoshi S, Kawaoka Y. 2019. Identification of Amino Acid Residues in Influenza A Virus PA-X That Contribute to Enhanced Shutoff Activity. Front Microbiol 10:432.

10. Oishi K, Yamayoshi S, Kawaoka Y. 2015. Mapping of a Region of the PA-X Protein of Influenza A Virus That Is Important for Its Shutoff Activity. J Virol 89:8661–8665.

11. Sun Y, Hu Z, Zhang X, Chen M, Wang Z, Xu G, Bi Y, Tong Q, Wang M, Sun H, Pu J, Iqbal M, Liu J. 2020. An R195K Mutation in the PA-X Protein Increases the Virulence and Transmission of Influenza A Virus in Mammalian Hosts. J Virol 94.

12. Chiem K, Lopez-Garcia D, Ortego J, Martinez-Sobrido L, DeDiego ML, Nogales A. 2022. Identification of Amino Acid Residues Required for Inhibition of Host Gene Expression by Influenza Virus A/Viet Nam/1203/2004 H5N1 PA-X. J Virol 96:e00408-21.
